# Supplementary material for: Genomic Analysis Highlights Putative Defective Susceptibility Genes in Tomato Germplasm
Source: Plants (Basel). 2023 Jun 12;12(12):2289. doi: 10.3390/plants12122289 (PMC10300993; doi:10.3390/plants12122289)
Supplement: Supplementary file 1 [file plants-12-02289-s001.zip › plants-2347108-Figure S1.pdf]

2.886.129; G>C; stop lost, splice variant

Deleterious SNP

Haplotypes	0: 2886129	1: 2886150	2: 2886166	3: 2886237	4: 2886304	5: 2886478	6: 2886592	7: 2886805	8: 2886951	9: 2887194	10: 2887462	11: 2887578	12: 2887635	13: 2887800	14: 2887876	15: 2887911	16: 2888036	17: 2888185	18: 2888261	19: 2888288	20: 2888299	21: 2888323	22: 2888359	23: 2888438	24: 2888446	25: 2888542
Cluster3:1(1,0)	G	T	T	C	G	T	C	A	A	G	A	C	Y	R	G	A	C	T	C	G	G	G	G	G	C	G
Cluster5:1(1,0)	G	T	T	C	G	T	C	G	A	G	A	C	T	G	G	A	C	T	C	G	G	G	G	G	C	T
Cluster15:1(1,0)	G	T	T	C	G	T	C	A	A	G	A	C	T	G	G	A	C	T	C	C	G	G	G	G	C	T
Cluster16:1(1,0)	G	T	T	C	G	T	C	G	A	C	T	G	T	G	G	A	C	T	C	G	G	G	G	G	C	T
Cluster9:1(1,0)	G	T	T	S	G	T	C	G	A	C	T	G	T	G	G	A	C	A	C	G	G	G	G	G	C	G
Cluster14:1(1,0)	G	T	A	C	G	T	C	G	A	C	T	G	T	G	G	A	C	T	C	G	G	A	G	G	C	T
Cluster20:1(1,0)	R	Y	W	C	G	Y	C	R	M	S	A	S	Y	R	S	R	C	A	C	G	G	G	G	C	T	G
Cluster8:1(1,0)	A	C	A	C	G	C	C	A	C	C	A	G	C	A	C	G	C	A	C	G	G	G	G	G	T	G
Cluster12:1(1,0)	A	C	A	C	G	C	C	A	M	S	A	G	C	A	C	G	C	A	C	G	G	G	G	C	T	G
Cluster11:1(1,0)	A	C	A	C	G	C	C	A	C	C	A	G	C	A	C	G	C	W	C	G	G	G	G	C	T	G
Cluster0:137(137,0)	A	C	A	C	G	C	C	A	C	C	A	G	C	A	C	G	C	A	C	G	G	G	G	C	T	G
Cluster4:1(1,0)	A	C	A	C	G	C	C	A	C	C	A	G	C	G	C	G	C	A	C	G	G	G	G	C	T	G
Cluster17:1(1,0)	A	C	A	C	G	C	C	A	C	C	A	G	Y	A	C	G	C	A	C	G	G	G	G	C	T	G
Cluster2:7(7,0)	A	C	A	C	G	C	C	A	C	C	A	G	C	A	C	G	C	A	C	G	G	G	R	C	T	G
Cluster7:1(1,0)	A	C	A	C	G	C	Y	A	C	C	A	G	C	A	C	G	C	A	C	G	G	G	G	C	T	G
Cluster10:1(1,0)	A	C	A	C	G	C	C	A	M	C	A	G	C	A	C	G	C	A	C	G	G	G	A	C	T	G
Cluster13:1(1,0)	A	C	A	C	G	C	C	A	C	C	A	G	C	A	C	G	Y	A	C	G	G	G	G	C	T	G
Cluster19:1(1,0)	A	C	A	C	G	Y	C	A	C	C	A	G	C	A	C	G	C	A	C	G	G	G	A	C	T	G
Cluster1:23(23,0)	A	C	A	C	G	C	C	A	C	C	A	G	C	A	C	G	C	A	C	G	G	G	A	C	T	G
Cluster18:1(1,0)	A	C	A	C	A	C	C	A	C	C	A	G	C	A	C	G	C	A	C	G	G	G	G	C	T	G
Cluster6:1(1,0)	A	C	A	C	G	C	C	A	C	C	A	G	C	A	C	G	C	A	Y	R	R	G	G	C	T	G

TS-068
TS-074
TS-186
TS-277
TS-409

BIK1	Solyc01g008860	SL2.50ch01	2886127	2888662

75.887.538; G>C; stop lost

Deleterious SNP

Haplotypes	0: 41233333	1: 41233798	2: 41234122	3: 41234229	4: 41234327	5: 41234490	6: 41234677	7: 41234699	8: 41235068	9: 41235319	10: 41235350	11: 41235439	12: 41235461
Cluster0:109(109,0)	G	T	C	C	C	A	T	G	G	A	G	C	G
Cluster7:1(1,0)	G	T	C	C	C	A	T	G	S	A	R	C	G
Cluster2:9(9,0)	G	T	C	C	C	A	T	S	G	A	G	C	G
Cluster3:3(3,0)	G	T	C	C	C	A	T	G	G	A	G	C	K
Cluster5:2(2,0)	G	T	C	C	C	A	T	G	G	R	G	C	G
Cluster8:1(1,0)	G	T	C	C	C	R	T	G	G	A	G	C	G
Cluster9:1(1,0)	G	T	C	C	C	A	T	G	G	A	G	Y	G
Cluster1:52(52,0)	G	T	C	C	C	A	T	C	G	A	G	C	G
Cluster13:1(1,0)	G	Y	C	Y	C	A	T	G	G	A	G	C	G
Cluster6:1(1,0)	G	T	C	Y	C	A	T	C	G	A	G	C	G
Cluster10:1(1,0)	G	Y	C	C	C	A	T	C	G	A	G	C	G
Cluster11:1(1,0)	A	T	C	C	C	R	K	G	G	R	R	C	K
Cluster12:1(1,0)	A	C	M	T	C	G	G	S	G	R	A	C	K
Cluster4:2(2,0)	A	C	A	T	T	G	G	G	G	G	A	C	T

TS-245

BIK1 Solyc01g028830 SL2.50ch01 41232211 41236033

75.887.538; A>G; stop lost

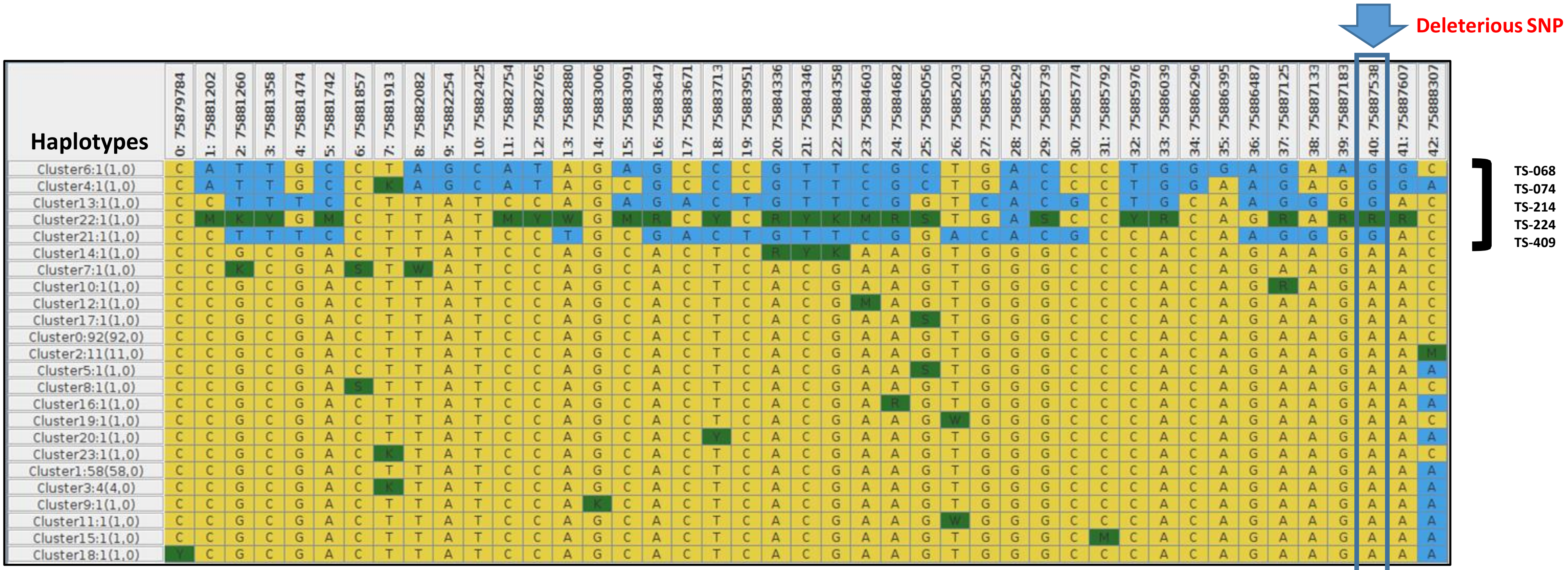

BIK1	Solyc01g067400	SL2.50ch01	75879603	75889164

49.186.199; G>A; stop gained

Deleterious SNP

Haplotypes	0: 49184258	1: 49184342	2: 49184367	3: 49184630	4: 49184891	5: 49184932	6: 49184997	7: 49185321	8: 49185629	9: 49186199	10: 49186237	11: 49186245	12: 49186384	13: 49186386	14: 49186610
Cluster1:14(14,0)	C	A	C	G	T	A	T	G	T	A	G	T	A	G	G
Cluster4:2(2,0)	C	A	C	G	T	A	T	G	T	A	G	T	A	G	R
Cluster16:1(1,0)	C	A	M	G	T	A	T	G	T	A	G	T	A	G	G
Cluster26:1(1,0)	C	A	C	G	T	A	T	G	T	R	G	T	A	G	G
Cluster31:1(1,0)	M	A	C	G	T	A	T	G	T	A	G	T	A	G	G
Cluster2:3(3,0)	C	A	C	G	T	A	T	G	T	A	G	T	A	G	A
Cluster9:1(1,0)	C	A	C	G	T	A	T	G	A	A	G	T	A	G	G
Cluster24:1(1,0)	C	A	C	G	T	A	T	G	T	A	G	T	G	G	G
Cluster25:1(1,0)	C	A	C	G	T	A	T	G	T	G	G	T	A	G	G
Cluster28:1(1,0)	C	A	C	G	T	A	G	G	T	A	G	T	A	G	G
Cluster12:1(1,0)	C	A	C	G	T	A	T	G	T	A	G	T	R	R	R
Cluster6:1(1,0)	C	A	C	C	T	A	T	G	T	A	G	T	G	G	G
Cluster13:1(1,0)	C	A	C	C	T	A	T	G	T	A	G	T	A	G	A
Cluster15:1(1,0)	M	A	C	G	T	A	K	R	W	A	G	T	A	G	G
Cluster8:1(1,0)	A	T	A	G	T	A	T	G	T	A	G	T	A	G	G
Cluster21:1(1,0)	C	A	C	G	T	A	G	G	T	A	G	T	R	A	A
Cluster5:2(2,0)	M	W	M	S	K	A	K	R	W	R	G	T	A	G	G
Cluster30:1(1,0)	A	A	C	S	K	A	K	R	W	R	G	T	A	G	R
Cluster20:1(1,0)	C	W	M	S	G	A	G	R	A	R	G	T	A	G	G
Cluster22:1(1,0)	M	W	M	S	T	A	G	R	W	R	G	T	R	G	R
Cluster27:1(1,0)	A	T	A	S	G	A	K	A	W	G	G	T	A	G	G
Cluster7:1(1,0)	A	T	M	C	K	A	G	A	A	G	G	T	A	G	G
Cluster10:1(1,0)	A	T	A	C	G	A	G	A	W	G	G	T	A	G	G
Cluster14:1(1,0)	M	T	A	C	G	A	G	A	A	G	G	T	A	G	G
Cluster17:1(1,0)	A	W	A	C	G	A	G	A	A	G	G	T	A	G	G
Cluster19:1(1,0)	A	T	A	C	G	A	K	A	A	G	G	T	A	G	G
Cluster23:1(1,0)	A	T	A	C	G	A	G	R	A	G	G	T	A	G	G
Cluster29:1(1,0)	A	T	A	C	G	A	G	A	A	R	G	T	A	G	G
Cluster0:135(135,0)	A	T	A	C	G	A	G	A	A	G	G	T	A	G	G
Cluster3:2(2,0)	A	T	A	C	G	A	G	A	A	G	G	T	A	R	G
Cluster18:1(1,0)	A	T	A	C	G	R	G	A	A	G	G	T	A	G	G
Cluster11:1(1,0)	A	T	A	C	G	A	G	A	A	G	K	Y	A	G	G
Cluster32:1(1,0)	A	T	A	C	G	A	G	A	A	G	G	T	A	A	G

TS-003
TS-004
TS-068
TS-078
TS-085
TS-090
TS-113
TS-121
TS-150
TS-186
TS-190
TS-214
TS-224
TS-245
TS-296

BIK1	Solyc04g050970	SL2.50ch04	49184225	49186758

1.640.061; T>G; splice variant

Deleterious SNP

Haplotypes	0: 1637986	1: 1638911	2: 1639335	3: 1639504	4: 1640061	5: 1640073	6: 1640805	7: 1641155	8: 1641412	9: 1642869	10: 1642931	11: 1642939
Cluster0:168(168,0)	T	C	G	C	T	T	A	A	A	G	A	C
Cluster1:6(6,0)	T	C	G	C	K	T	A	A	A	G	A	C
Cluster2:1(1,0)	Y	C	G	C	T	T	A	A	A	G	A	C
Cluster3:1(1,0)	T	C	S	C	T	T	A	A	A	G	A	C
Cluster4:1(1,0)	T	C	G	C	T	T	A	A	R	G	A	C
Cluster5:1(1,0)	T	Y	G	C	T	T	A	A	A	G	A	C
Cluster6:1(1,0)	T	C	G	C	K	T	A	W	A	G	A	C
Cluster7:1(1,0)	T	C	G	C	T	T	A	A	A	G	A	Y
Cluster8:1(1,0)	T	C	G	Y	T	T	A	A	A	G	A	C
Cluster9:1(1,0)	T	C	G	C	T	T	A	A	A	S	A	C
Cluster10:1(1,0)	T	C	G	C	T	T	W	A	A	G	A	C
Cluster11:1(1,0)	T	C	G	C	T	T	A	A	A	G	G	T
Cluster12:1(1,0)	T	C	G	C	T	Y	A	A	A	G	A	C

TS-005
TS-008
TS-011
TS-012
TS-245

**BIK1**	**Solyc05g007050**	**SL2.50ch05**	**1637910**	**1643930**

31.013.858; A>T; stop gained

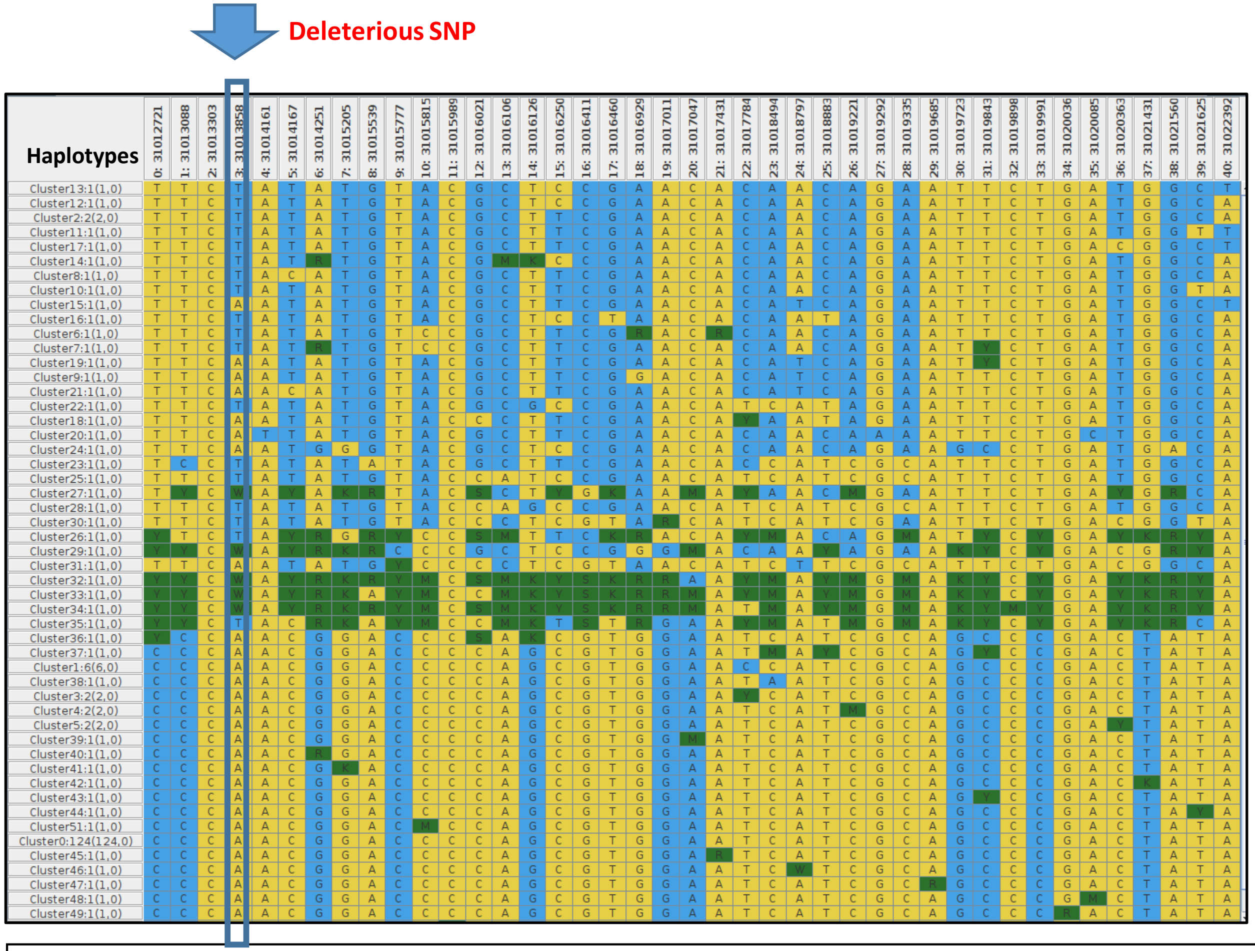

TS-003
TS-008
TS-085
TS-108
TS-186
TS-190
TS-277

BIK1	Solyc05g024290	SL2.50ch05	31012552	31022465

527.016; T>G; splice variant

Deleterious SNP

Haplotypes	0: 526215	1: 526700	2: 526839	3: 526918	4: 527016	5: 527032	6: 527035	7: 527156	8: 527216	9: 527287	10: 527290	11: 527321	12: 527429	13: 527459	14: 527585	15: 527716	16: 527836	17: 527861
Cluster5:2(2,0)	G	A	G	T	G	G	T	C	C	A	A	A	C	A	C	T	T	A
Cluster7:1(1,0)	G	A	G	T	K	G	T	C	C	A	A	A	C	A	C	T	T	A
Cluster0:97(97,0)	G	A	G	T	T	G	T	C	C	A	A	A	C	A	C	T	T	A
Cluster3:8(8,0)	G	A	G	T	T	G	T	C	C	A	A	A	Y	A	C	T	T	A
Cluster4:2(2,0)	G	A	G	T	T	R	T	C	C	A	A	A	C	A	C	T	T	A
Cluster6:2(2,0)	G	A	G	T	T	G	T	C	C	A	A	A	C	A	C	T	T	A
Cluster9:1(1,0)	G	A	G	T	T	G	T	C	C	A	A	A	C	A	C	T	T	A
Cluster10:1(1,0)	G	A	G	T	T	G	T	C	C	A	A	A	C	A	Y	T	T	A
Cluster24:1(1,0)	G	A	G	T	K	G	T	C	C	A	A	A	C	A	C	T	T	A
Cluster25:1(1,0)	G	A	G	T	T	G	T	C	C	A	A	A	C	R	C	T	T	A
Cluster26:1(1,0)	G	A	G	T	T	G	T	C	C	A	W	A	C	A	C	T	T	A
Cluster1:42(42,0)	G	A	G	T	T	G	T	C	C	A	A	A	T	A	C	T	T	A
Cluster2:11(11,0)	G	A	G	T	T	G	T	C	C	A	A	A	C	A	C	T	T	A
Cluster12:1(1,0)	G	A	G	T	T	G	T	C	C	A	A	A	C	A	C	T	T	A
Cluster14:1(1,0)	G	A	G	T	T	A	T	C	C	A	A	A	C	A	C	T	T	A
Cluster17:1(1,0)	G	A	G	T	T	G	T	C	C	A	A	R	C	A	C	T	K	A
Cluster16:1(1,0)	G	R	G	T	T	G	T	C	C	A	A	A	T	A	C	T	T	A
Cluster8:1(1,0)	G	A	G	T	T	G	T	C	C	A	A	A	T	A	C	T	T	A
Cluster19:1(1,0)	K	A	R	Y	T	G	T	M	C	A	A	A	C	A	C	W	T	R
Cluster23:1(1,0)	G	A	G	T	T	G	T	C	C	A	T	A	C	A	C	T	T	A
Cluster15:1(1,0)	G	A	G	T	T	G	T	M	C	A	A	R	C	A	C	W	K	R
Cluster20:1(1,0)	G	A	R	Y	T	G	C	M	C	A	A	R	C	A	C	W	T	A
Cluster21:1(1,0)	K	A	R	Y	T	G	C	M	C	A	A	R	C	A	C	W	T	R
Cluster18:1(1,0)	K	A	R	C	T	G	C	M	C	A	A	R	C	A	C	W	T	R
Cluster13:1(1,0)	G	R	R	T	T	G	T	M	C	A	A	R	Y	A	C	W	G	R
Cluster22:1(1,0)	G	A	A	T	T	G	T	C	C	G	T	G	C	A	T	A	T	G
Cluster11:1(1,0)	T	A	A	T	T	G	T	C	G	G	T	G	C	A	T	A	T	A
Cluster27:1(1,0)	T	G	A	T	T	G	C	A	G	G	T	G	C	A	C	A	G	G

Haplotypes	18: 527933	19: 527966	20: 528020	21: 528042	22: 528080	23: 528111	24: 528206	25: 528260	26: 528370	27: 528404	28: 528589	29: 528634	30: 528656	31: 528716	32: 528745	33: 528829	34: 528919
Cluster5:2(2,0)	C	C	T	C	T	A	A	C	T	C	T	G	C	C	T	A	T
Cluster7:1(1,0)	C	C	T	C	T	A	A	C	T	C	T	G	C	C	T	A	T
Cluster0:97(97,0)	C	C	T	C	T	A	A	C	T	C	T	G	C	C	T	A	T
Cluster3:8(8,0)	C	C	T	C	T	A	A	C	T	C	T	G	C	C	T	A	T
Cluster4:2(2,0)	C	C	T	C	T	A	A	C	T	C	T	G	C	C	T	A	T
Cluster6:2(2,0)	C	C	T	C	T	A	A	C	T	C	T	G	C	C	T	A	Y
Cluster9:1(1,0)	C	C	T	C	T	A	A	C	T	M	T	G	C	C	T	A	T
Cluster10:1(1,0)	C	C	T	C	T	A	A	C	T	C	T	G	C	C	T	A	T
Cluster24:1(1,0)	C	C	T	C	T	A	A	C	T	A	T	G	C	C	T	A	T
Cluster25:1(1,0)	C	C	T	C	T	A	A	C	T	C	T	G	C	C	T	A	T
Cluster26:1(1,0)	C	C	T	C	T	A	A	C	T	C	T	G	C	C	T	A	T
Cluster1:42(42,0)	C	C	T	C	T	A	A	C	T	C	T	G	C	C	T	A	T
Cluster2:11(11,0)	C	C	T	C	T	A	A	C	T	A	T	G	C	C	T	A	T
Cluster12:1(1,0)	C	C	Y	Y	T	A	A	C	T	C	T	G	C	C	T	A	T
Cluster14:1(1,0)	C	C	T	C	T	A	A	C	T	C	T	G	C	C	T	A	T
Cluster17:1(1,0)	C	C	T	C	T	A	A	C	T	C	T	G	C	C	T	A	T
Cluster16:1(1,0)	C	C	T	C	T	A	A	C	T	C	T	G	C	C	T	A	T
Cluster8:1(1,0)	C	C	T	C	T	A	A	S	T	C	W	G	Y	C	T	A	T
Cluster19:1(1,0)	C	C	T	C	T	A	A	C	Y	C	T	K	Y	M	K	A	Y
Cluster23:1(1,0)	C	C	C	C	T	A	A	G	T	C	A	G	T	C	T	A	C
Cluster15:1(1,0)	Y	M	Y	Y	W	M	W	C	T	C	T	G	C	C	T	R	T
Cluster20:1(1,0)	C	C	T	C	T	A	A	C	Y	C	T	K	Y	M	K	A	Y
Cluster21:1(1,0)	C	C	T	C	T	A	A	C	T	C	T	K	Y	M	K	A	T
Cluster18:1(1,0)	C	M	T	C	T	A	A	C	Y	C	T	K	Y	M	K	A	Y
Cluster13:1(1,0)	Y	M	Y	Y	W	M	W	C	T	C	T	K	C	C	K	A	T
Cluster22:1(1,0)	C	A	C	T	T	A	A	G	C	C	T	G	T	C	T	A	C
Cluster11:1(1,0)	C	A	C	T	T	A	A	G	C	C	T	G	T	C	T	A	C
Cluster27:1(1,0)	T	A	C	T	A	C	T	G	C	C	T	T	T	C	G	G	T

TS-004
TS-005

BIK1 Solyc06g005500 SL2.50ch06 525975 529157

39.717.979; T>A; stop lost

Deleterious SNP

Haplotypes	0: 39716951	1: 39717011	2: 39717410	3: 39717652	4: 39717864	5: 39717979	6: 39718094	7: 39718132	8: 39718162	9: 39718168	10: 39718819	11: 39718853	12: 39718884	
Cluster0:178(178,0)	A	C	T	T	C	T	G	A	T	T	A	G	T	
Cluster1:1(1,0)	A	T	T	A	C	A	C	A	T	T	C	T	T	TS-074
Cluster2:1(1,0)	A	T	T	A	C	T	C	A	T	C	A	G	T	
Cluster3:1(1,0)	A	T	T	A	C	T	C	A	A	T	C	G	A	
Cluster4:1(1,0)	A	T	T	A	A	T	C	A	A	T	C	G	T	
Cluster5:1(1,0)	A	C	T	A	A	T	G	A	T	T	C	G	T	
Cluster6:1(1,0)	A	Y	T	W	C	W	S	A	T	T	M	K	T	TS-068
Cluster7:1(1,0)	A	Y	T	A	C	T	S	A	T	C	C	G	A	

**BIK1**	**Solyc06g062920**	**SL2.50ch06**	**39716883**	**39719077**

48.908.349; T>A; stop gained

Deleterious SNP

Haplotypes	0: 48904895	1: 48904949	2: 48905017	3: 48905023	4: 48905053	5: 48905123	6: 48905209	7: 48905423	8: 48905455	9: 48905458	10: 48905551	11: 48905575	12: 48905704	13: 48905715	14: 48905726	15: 48905728	16: 48905941	17: 48906237
Cluster5:1(1,0)	G	C	A	A	A	C	T	G	T	T	C	C	C	T	A	T	T	A
Cluster8:1(1,0)	G	C	G	G	G	T	C	G	T	C	C	C	C	C	A	T	A	A
Cluster11:1(1,0)	G	C	G	G	G	Y	C	G	T	C	T	C	T	T	A	T	T	C
Cluster0:174(174,0)	G	C	G	G	G	T	C	G	T	C	T	C	T	T	A	T	T	C
Cluster6:1(1,0)	G	Y	G	G	G	T	C	G	T	C	T	C	T	T	A	T	T	C
Cluster7:1(1,0)	G	C	G	G	G	T	C	G	T	C	T	C	T	T	A	T	T	C
Cluster10:1(1,0)	G	C	G	G	G	T	C	R	T	C	T	C	T	T	A	T	T	C
Cluster1:1(1,0)	G	C	G	G	G	T	C	G	T	C	T	C	T	T	A	T	T	C
Cluster2:1(1,0)	R	C	G	G	G	T	C	G	T	C	T	C	T	T	A	T	T	M
Cluster4:1(1,0)	A	C	G	G	G	C	C	G	G	C	C	G	C	C	T	A	W	A
Cluster9:1(1,0)	A	C	G	G	G	C	C	G	G	C	C	G	T	T	A	A	W	A
Cluster3:1(1,0)	A	C	G	G	G	C	C	G	G	C	C	G	C	C	W	W	A	A

Haplotypes	18: 48906249	19: 48906267	20: 48906293	21: 48906297	22: 48906372	23: 48906848	24: 48907068	25: 48907197	26: 48907312	27: 48907369	28: 48907402	29: 48907904	30: 48907929	31: 48907952	32: 48907983	33: 48908299	34: 48908317	35: 48908349
Cluster5:1(1,0)	C	G	A	C	A	C	G	T	T	C	G	C	G	T	A	T	T	A
Cluster8:1(1,0)	C	G	T	T	A	C	G	T	T	T	A	C	G	T	A	T	T	A
Cluster11:1(1,0)	C	G	A	C	A	C	G	T	T	C	G	G	C	C	T	T	C	T
Cluster0:174(174,0)	C	G	A	C	A	C	G	T	T	C	G	G	C	C	T	T	C	T
Cluster6:1(1,0)	C	G	A	C	A	C	G	T	T	C	G	G	C	C	T	T	C	T
Cluster7:1(1,0)	C	G	A	C	A	C	R	T	T	C	G	G	C	C	T	T	C	T
Cluster10:1(1,0)	C	G	A	C	A	C	G	T	T	C	G	G	C	C	T	T	C	T
Cluster1:1(1,0)	C	G	W	C	R	C	G	T	T	C	G	G	C	C	T	T	C	T
Cluster2:1(1,0)	M	G	W	C	R	C	G	T	T	C	G	G	C	C	T	T	C	T
Cluster4:1(1,0)	A	G	A	C	R	T	G	G	T	C	G	G	C	C	T	C	C	T
Cluster9:1(1,0)	A	A	T	C	G	Y	G	G	C	C	G	G	C	C	T	T	C	T
Cluster3:1(1,0)	A	A	T	C	G	T	G	G	C	C	G	G	C	C	T	C	C	T

TS-224

**BIK1**	**Solyc06g083500**	**SL2.50ch06**	**48904883**	**48908656**

61.543.477; C>T; stop gained

Deleterious SNP

Haplotypes	0: 61542107	1: 61542207	2: 61542277	3: 61542281	4: 61542455	5: 61542474	6: 61542630	7: 61542641	8: 61542651	9: 61542683	10: 61542850	11: 61542876	12: 61542970	13: 61543029	14: 61543102	15: 61543350	16: 61543467	17: 61543477	18: 61543522	19: 61543586	20: 61543646	21: 61543690	22: 61543730	23: 61543890	24: 61543960	25: 61544038	26: 61544048	27: 61544230	28: 61544393	29: 61544517	30: 61544579	31: 61544673	32: 61544723	33: 61544770	34: 61544805	35: 61544911	36: 61545017	
Cluster0:139(139,0)	T	C	C	G	T	C	T	G	G	A	G	G	G	G	A	C	C	C	G	A	T	A	T	T	T	A	A	G	G	C	C	C	A	C	T	C	C	
Cluster1:15(15,0)	T	C	C	G	T	C	T	G	G	A	G	G	G	G	A	C	C	C	G	A	T	A	T	T	T	A	A	G	G	C	C	C	A	C	C	C	C	
Cluster2:4(4,0)	T	C	C	G	T	C	T	G	G	A	G	G	G	G	A	C	C	C	G	A	T	A	T	T	T	A	A	G	G	C	C	C	A	C	Y	C	C	
Cluster3:1(1,0)	T	C	C	G	T	C	T	G	G	A	G	G	G	G	A	C	C	C	G	A	T	A	T	T	T	A	A	G	G	C	C	C	M	C	T	C	C	
Cluster4:1(1,0)	T	C	C	G	T	C	T	G	G	A	G	G	G	G	A	C	C	C	G	R	T	A	T	T	T	A	A	G	G	C	C	C	A	C	T	C	C	
Cluster5:1(1,0)	T	Y	C	G	W	Y	Y	R	G	W	G	R	G	R	W	Y	C	Y	R	R	W	M	W	Y	K	A	A	R	G	Y	Y	S	M	Y	Y	M	Y	TS-11
Cluster9:1(1,0)	T	C	C	G	T	C	T	G	G	A	G	G	G	G	A	C	C	C	G	A	T	A	T	T	T	A	A	G	G	Y	C	C	A	C	T	C	C	
Cluster13:1(1,0)	T	Y	C	G	T	C	T	G	G	A	G	G	G	G	A	C	C	C	G	A	T	A	T	T	T	A	A	G	G	C	C	C	A	C	T	C	C	
Cluster22:1(1,0)	T	C	C	G	T	C	T	G	G	A	G	G	G	G	A	C	C	C	G	A	T	A	T	T	T	A	A	R	G	C	C	C	A	C	T	C	C	
Cluster26:1(1,0)	T	C	C	G	T	C	T	G	G	A	G	G	G	G	A	C	C	Y	G	A	T	A	T	T	T	A	A	G	G	C	C	C	A	C	T	C	C	TS-12
Cluster27:1(1,0)	T	C	C	G	T	C	T	G	G	A	G	G	G	G	A	C	C	C	G	A	T	A	T	T	K	A	A	G	G	C	C	C	A	C	T	C	Y	
Cluster28:1(1,0)	T	C	C	G	T	C	T	G	G	A	G	G	G	G	A	Y	C	C	G	A	T	M	T	T	T	A	A	G	G	C	C	C	A	C	T	C	C	
Cluster21:1(1,0)	Y	Y	C	G	W	Y	T	G	G	W	G	R	R	R	W	Y	C	Y	R	R	W	M	W	Y	K	A	A	R	G	Y	Y	S	M	Y	Y	M	Y	
Cluster24:1(1,0)	T	C	C	G	T	C	T	G	G	A	C	G	G	G	A	C	C	C	G	A	T	A	T	T	T	A	A	G	G	C	C	C	A	C	T	C	C	
Cluster7:1(1,0)	T	C	C	G	W	Y	Y	R	R	W	G	R	R	R	W	Y	C	Y	R	A	W	M	W	Y	T	A	W	G	G	Y	Y	S	M	C	Y	M	Y	
Cluster6:1(1,0)	Y	C	S	R	T	C	T	G	G	W	G	R	G	G	W	Y	Y	Y	R	R	W	M	W	Y	K	A	A	G	G	Y	Y	S	M	Y	Y	M	Y	
Cluster29:1(1,0)	T	Y	S	R	W	Y	Y	R	R	W	G	R	R	R	W	Y	C	Y	R	R	W	M	W	Y	K	A	A	R	G	Y	Y	S	M	Y	Y	C	Y	
Cluster14:1(1,0)	T	Y	C	G	W	Y	Y	R	R	W	G	R	R	R	W	Y	Y	Y	R	R	W	M	W	T	K	R	W	G	G	C	C	S	A	C	T	M	Y	
Cluster20:1(1,0)	Y	C	S	G	W	Y	T	G	R	W	G	R	R	R	W	Y	C	Y	R	R	W	M	W	Y	K	A	W	G	G	Y	Y	S	M	Y	Y	M	Y	
Cluster8:1(1,0)	Y	Y	S	R	W	Y	Y	R	R	W	G	R	R	G	W	Y	Y	C	G	R	W	M	W	Y	K	A	A	R	G	C	C	S	M	Y	Y	M	Y	
Cluster10:1(1,0)	T	C	S	G	W	C	Y	R	R	W	G	G	R	R	W	Y	Y	Y	R	R	T	M	T	Y	K	A	W	R	K	Y	Y	S	M	C	Y	C	Y	
Cluster18:1(1,0)	Y	Y	S	R	W	Y	Y	R	R	W	G	G	R	R	W	Y	Y	C	R	R	W	M	W	Y	K	A	A	R	G	C	Y	S	M	C	T	C	Y	
Cluster19:1(1,0)	Y	C	S	R	W	Y	T	G	G	W	G	G	R	R	W	Y	Y	C	R	R	W	M	T	T	K	A	W	G	G	Y	Y	S	M	Y	T	M	Y	
Cluster23:1(1,0)	Y	Y	S	R	W	Y	Y	R	R	W	G	R	R	G	W	Y	Y	Y	R	R	W	M	W	T	K	A	A	R	G	Y	Y	S	M	Y	C	M	Y	
Cluster11:1(1,0)	Y	Y	S	R	W	Y	Y	R	R	W	G	G	R	R	W	Y	Y	Y	R	R	W	M	W	Y	K	A	W	R	G	Y	Y	S	M	Y	Y	M	Y	
Cluster12:1(1,0)	Y	Y	S	G	W	Y	Y	R	R	W	G	R	R	R	W	Y	Y	C	R	R	W	M	W	Y	K	A	W	R	K	Y	Y	S	M	Y	C	M	Y	
Cluster25:1(1,0)	Y	C	S	R	T	C	Y	G	R	W	G	R	R	R	W	Y	Y	Y	R	R	W	M	W	Y	K	A	W	R	G	Y	Y	S	M	Y	Y	M	Y	
Cluster15:1(1,0)	Y	Y	S	R	W	Y	Y	R	R	W	G	R	R	G	W	Y	Y	Y	R	R	W	M	W	Y	K	R	W	R	G	Y	Y	S	M	C	Y	M	Y	
Cluster16:1(1,0)	Y	Y	S	R	W	C	Y	R	R	W	G	R	R	R	W	Y	Y	Y	R	R	W	M	W	Y	K	A	W	G	K	Y	Y	S	M	Y	Y	M	Y	
Cluster17:1(1,0)	T	Y	S	R	T	C	Y	R	R	W	G	G	R	R	W	Y	Y	Y	G	R	W	M	T	Y	K	R	W	R	K	Y	Y	S	M	Y	Y	M	Y	

BIK1	Solyc08g077560	SL2.50ch08	61541667	61545970

2.414.006; C>T; stop gained

Deleterious SNP

Haplotypes	0: 2412901	1: 2412912	2: 2413087	3: 2413215	4: 2413514	5: 2413585	6: 2413741	7: 2413886	8: 2414006
Cluster1:58(58,0)	C	G	T	A	A	T	G	A	T
Cluster2:9(9,0)	C	G	T	A	A	T	G	A	Y
Cluster3:1(1,0)	C	G	T	A	A	T	G	R	T
Cluster0:108(108,0)	C	G	T	A	A	T	G	A	C
Cluster5:1(1,0)	C	G	T	A	A	T	T	A	T
Cluster6:1(1,0)	C	G	T	A	A	Y	G	A	Y
Cluster11:1(1,0)	C	G	T	A	A	T	G	G	T
Cluster12:1(1,0)	C	G	T	A	A	Y	G	R	T
Cluster4:1(1,0)	C	G	T	A	R	T	G	A	C
Cluster8:1(1,0)	C	G	T	A	A	T	G	R	C
Cluster9:1(1,0)	M	R	T	A	A	T	G	R	T
Cluster7:1(1,0)	C	G	C	A	A	T	T	R	T
Cluster10:1(1,0)	A	A	T	G	A	C	G	G	T

TS-004
TS-005
TS-007
TS-068
TS-074
TS-078
TS-089
TS-090
TS-113
TS-122
TS-127
TS-135
TS-150
TS-224
TS-245
TS-277
TS-409

**DMR1**	**Solyc04g008760**	**SL2.50ch04**	**2412878**	**2414011**

52.083.445; A>G; stop lost

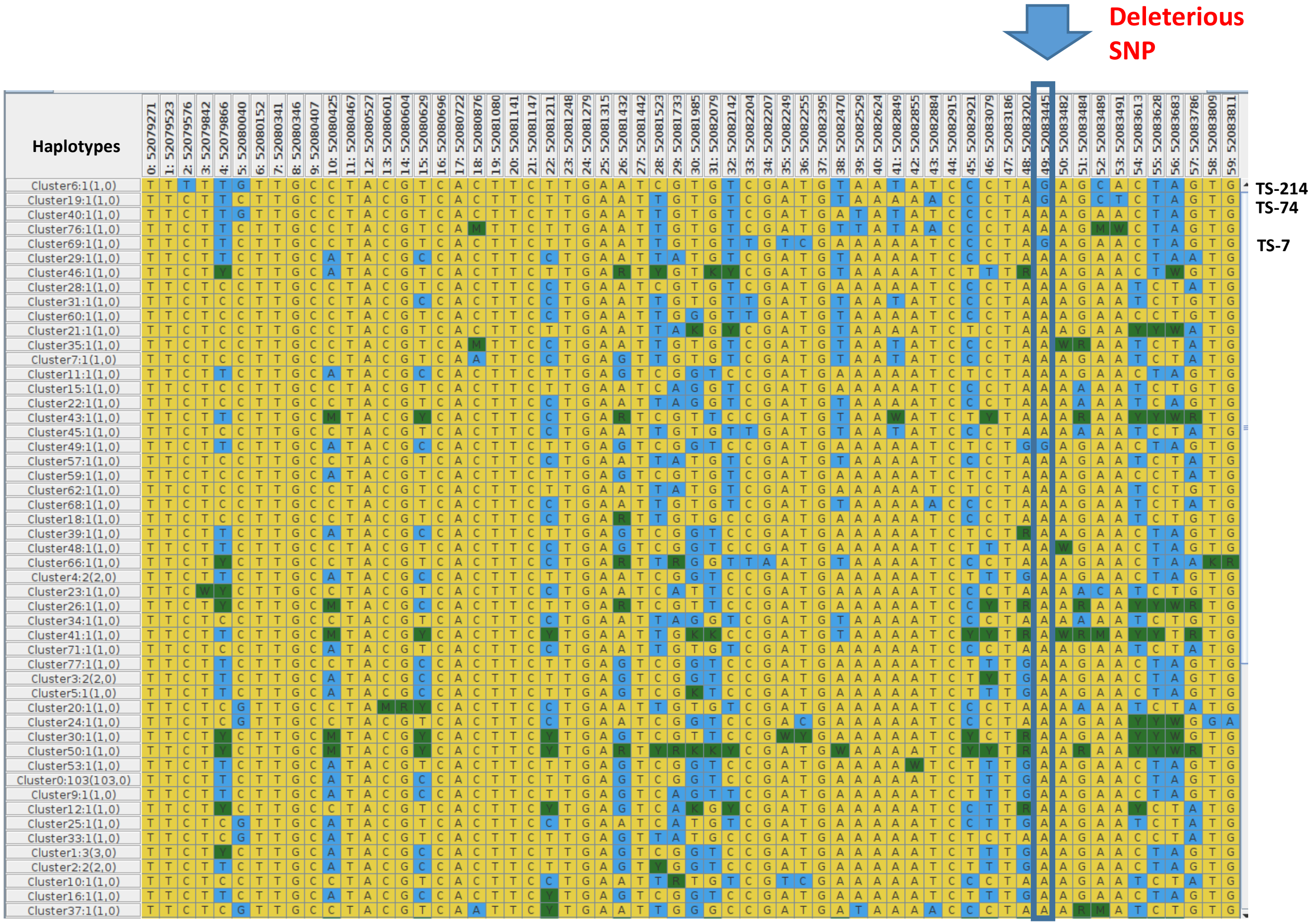

DMR6	Solyc03g080190	SL2.50ch03	52078938	52083877

50.610.274; A>T; stop gained

Deleterious SNP

Haplotypes	0: 50608525	1: 50609060	2: 50609278	3: 50609299	4: 50609372	5: 50609571	6: 50609995	7: 50610274	8: 50610370	9: 50610420	10: 50610609	11: 50610749	12: 50610751
Cluster9:1(1,0)	T	A	T	G	G	A	T	T	C	A	T	C	A
Cluster3:1(1,0)	T	A	T	G	G	A	T	T	C	A	T	C	A
Cluster12:1(1,0)	T	A	T	G	G	A	T	T	C	A	T	C	G
Cluster8:1(1,0)	T	A	T	G	G	A	T	T	C	A	T	C	A
Cluster11:1(1,0)	C	A	T	G	G	G	C	A	M	R	G	C	A
Cluster14:1(1,0)	C	A	T	G	G	R	C	A	A	G	K	C	A
Cluster16:1(1,0)	C	A	T	G	G	G	Y	A	A	R	G	C	A
Cluster17:1(1,0)	C	A	T	G	G	G	C	A	A	G	G	C	A
Cluster1:2(2,0)	C	A	T	G	G	G	C	A	A	G	G	C	A
Cluster2:2(2,0)	C	A	T	G	G	G	C	A	A	G	G	C	A
Cluster5:1(1,0)	Y	A	T	G	G	G	C	A	A	G	G	C	A
Cluster0:166(166,0)	C	A	T	G	G	G	C	A	A	G	G	C	A
Cluster6:1(1,0)	C	A	Y	K	G	G	Y	A	A	G	G	C	A
Cluster7:1(1,0)	C	A	T	G	G	G	C	A	A	G	G	C	A
Cluster15:1(1,0)	C	A	T	G	G	G	C	A	A	G	G	C	A
Cluster4:1(1,0)	C	R	T	K	R	G	C	A	A	G	G	C	A
Cluster13:1(1,0)	C	A	T	G	G	G	C	A	A	G	G	C	A
Cluster10:1(1,0)	C	A	T	G	G	G	C	A	A	G	G	G	A

Haplotypes	13: 50610859	14: 50610947	15: 50611053	16: 50611148	17: 50611377	18: 50611528	19: 50611651	20: 50611730	21: 50611813	22: 50611817	23: 50611887	24: 50611910
Cluster9:1(1,0)	C	C	T	A	C	A	A	T	A	G	T	C
Cluster3:1(1,0)	C	C	T	A	C	A	A	A	A	G	T	C
Cluster12:1(1,0)	C	C	T	W	C	A	A	T	A	G	W	C
Cluster8:1(1,0)	T	T	T	A	C	A	A	A	A	G	T	C
Cluster11:1(1,0)	T	T	T	T	T	G	A	A	G	G	T	C
Cluster14:1(1,0)	T	T	T	T	T	R	A	A	G	G	W	C
Cluster16:1(1,0)	T	T	T	T	T	G	A	A	G	G	T	C
Cluster17:1(1,0)	T	T	T	T	C	G	A	A	G	G	T	C
Cluster1:2(2,0)	T	T	T	T	Y	G	A	A	G	G	T	C
Cluster2:2(2,0)	T	T	T	T	T	G	A	W	G	G	T	C
Cluster5:1(1,0)	T	T	T	T	T	G	A	A	G	G	T	C
Cluster0:166(166,0)	T	T	T	T	T	G	A	A	G	G	T	C
Cluster6:1(1,0)	T	T	T	T	T	G	A	W	G	G	T	C
Cluster7:1(1,0)	T	T	Y	T	T	G	A	A	G	G	T	C
Cluster15:1(1,0)	T	T	T	T	T	G	A	A	G	R	T	C
Cluster4:1(1,0)	T	T	T	T	T	G	A	W	G	G	T	C
Cluster13:1(1,0)	T	T	T	T	C	G	G	A	G	G	T	T
Cluster10:1(1,0)	T	T	T	T	C	G	G	A	G	G	T	T

TS-89
TS-135
TS-150

DND1	Solyc02g088560	SL2.50ch02	50608396	50612219

42.468.063; G>T; intron variant

42.471.760; G>A; stop gained

Deleterious SNP (5: 42468063)

Deleterious SNP (38: 42471760)

Haplotypes	0: 42467302	1: 42467440	2: 42467523	3: 42467756	4: 42468050	5: 42468063	6: 42468109	7: 42468182	8: 42468196	9: 42468287	10: 42468574	11: 42468602	12: 42468624	13: 42468729	14: 42468734	15: 42469211	16: 42469340	17: 42469383	18: 42469713
Cluster0:77(77,0)	T	G	T	C	A	G	C	C	T	T	T	A	A	C	A	T	C	A	C
Cluster17:1(1,0)	T	G	T	M	A	G	C	C	T	T	T	A	A	C	A	T	C	A	C
Cluster23:1(1,0)	T	G	T	C	A	G	C	C	T	T	T	A	A	C	A	T	C	A	C
Cluster1:57(57,0)	T	G	T	C	A	G	C	C	T	T	T	A	A	C	A	T	C	A	C
Cluster4:1(1,0)	T	G	T	C	A	G	C	C	T	T	T	A	A	C	A	T	C	A	C
Cluster27:1(1,0)	T	G	T	C	A	G	C	C	T	T	W	A	A	C	A	T	C	A	C
Cluster30:1(1,0)	T	G	T	C	A	G	C	C	T	T	T	A	A	C	A	T	C	A	C
Cluster2:20(20,0)	T	G	T	C	A	G	C	C	T	T	T	A	A	C	A	T	C	A	C
Cluster3:1(1,0)	T	G	T	C	A	G	C	C	T	T	T	A	A	C	A	T	C	A	C
Cluster28:1(1,0)	Y	A	Y	C	A	G	C	C	T	T	T	A	A	C	A	T	C	A	C
Cluster14:1(1,0)	T	G	T	C	A	G	C	C	T	T	T	A	A	C	A	T	C	A	C
Cluster33:1(1,0)	T	G	T	C	A	G	C	C	T	T	T	A	A	C	A	T	C	A	C
Cluster8:1(1,0)	T	G	T	C	A	G	C	C	T	T	T	A	A	C	A	T	Y	G	C
Cluster13:1(1,0)	T	G	Y	M	A	K	Y	C	T	T	T	A	A	C	A	T	C	A	C
Cluster26:1(1,0)	C	A	C	A	A	T	T	C	T	T	T	A	A	C	A	G	C	A	C
Cluster12:1(1,0)	Y	G	C	A	A	K	Y	Y	T	W	W	A	R	C	A	T	C	A	C
Cluster11:1(1,0)	T	R	Y	C	A	T	Y	C	T	W	T	A	R	C	A	T	Y	G	T
Cluster24:1(1,0)	T	A	C	A	T	T	C	T	C	A	T	C	G	C	A	T	C	A	C
Cluster19:1(1,0)	Y	R	Y	C	A	T	Y	Y	T	W	W	A	R	C	A	T	Y	A	C
Cluster20:1(1,0)	Y	G	Y	M	A	K	Y	Y	T	W	W	A	R	Y	R	K	Y	R	Y
Cluster9:1(1,0)	C	G	C	C	A	T	T	C	T	T	T	A	G	T	G	G	C	G	C
Cluster7:1(1,0)	C	G	C	C	A	T	T	C	T	A	A	A	G	T	G	T	C	A	T
Cluster5:1(1,0)	C	A	C	A	A	T	T	T	T	A	A	A	G	T	G	G	T	A	T
Cluster29:1(1,0)	C	A	C	M	A	T	Y	Y	T	W	W	A	R	Y	R	G	C	A	T
Cluster15:1(1,0)	C	G	C	A	A	T	T	C	T	A	A	A	G	T	G	T	C	A	T
Cluster16:1(1,0)	C	A	C	A	A	T	T	T	T	A	A	A	G	T	G	T	T	A	T
Cluster31:1(1,0)	C	G	C	A	A	T	T	T	T	A	A	A	G	T	G	G	T	A	T
Cluster10:1(1,0)	T	A	C	C	A	T	T	C	T	A	A	A	G	T	G	T	T	A	C
Cluster18:1(1,0)	C	G	C	A	A	T	T	C	T	A	A	A	G	T	A	G	T	A	T
Cluster6:1(1,0)	C	A	C	A	A	T	T	C	T	A	W	A	G	T	G	G	T	G	T
Cluster25:1(1,0)	C	G	C	A	A	T	T	T	T	A	A	A	G	T	G	T	T	G	T
Cluster32:1(1,0)	C	A	C	A	A	T	T	T	T	A	A	A	G	T	G	T	T	G	T
Cluster22:1(1,0)	C	A	C	A	A	T	T	T	T	A	A	A	G	T	G	G	T	A	T
Cluster21:1(1,0)	C	R	C	A	A	T	T	T	T	A	A	A	G	T	G	G	T	G	T

Haplotypes	19: 42469805	20: 42469852	21: 42469880	22: 42470061	23: 42470214	24: 42470345	25: 42470449	26: 42470516	27: 42470584	28: 42470631	29: 42470636	30: 42470656	31: 42470665	32: 42470809	33: 42470811	34: 42470969	35: 42471590	36: 42471739	37: 42471746	38: 42471760
Cluster0:77(77,0)	G	G	A	T	C	G	C	A	A	T	T	T	C	A	C	C	T	A	A	G
Cluster17:1(1,0)	G	G	A	T	C	G	C	A	A	T	T	T	C	A	C	C	T	A	A	G
Cluster23:1(1,0)	R	G	A	T	C	G	C	A	A	T	T	T	C	A	C	C	T	A	A	G
Cluster1:57(57,0)	A	G	A	T	C	G	C	A	A	T	T	T	C	A	C	C	T	A	A	G
Cluster4:1(1,0)	G	G	A	T	C	G	C	A	A	T	T	A	C	A	C	C	T	A	A	G
Cluster27:1(1,0)	A	G	A	T	C	G	C	A	A	T	T	T	C	A	C	C	T	A	A	G
Cluster30:1(1,0)	A	G	A	T	C	G	C	A	A	T	T	W	C	A	C	C	T	A	A	G
Cluster2:20(20,0)	A	G	A	T	C	G	C	A	A	T	T	A	C	A	C	C	T	A	A	G
Cluster3:1(1,0)	A	G	A	T	C	G	C	A	A	T	T	T	C	A	C	A	T	A	A	G
Cluster28:1(1,0)	G	G	A	T	C	G	C	A	A	T	T	T	C	A	C	C	T	A	A	G
Cluster14:1(1,0)	G	G	A	T	C	G	C	A	A	T	T	T	C	A	C	C	T	C	T	A
Cluster33:1(1,0)	A	R	A	T	C	R	C	A	A	T	T	A	C	A	C	M	T	A	A	G
Cluster8:1(1,0)	A	G	A	T	C	G	C	R	R	Y	T	T	C	A	C	C	T	A	A	G
Cluster13:1(1,0)	A	R	R	T	C	G	C	R	A	T	T	T	C	A	C	C	T	A	A	R
Cluster26:1(1,0)	G	G	A	T	C	A	C	A	A	T	T	T	C	A	C	C	T	A	A	G
Cluster12:1(1,0)	A	R	R	W	Y	A	Y	R	R	T	Y	T	Y	A	C	M	T	A	A	G
Cluster11:1(1,0)	A	R	R	T	C	R	T	A	A	Y	Y	W	Y	A	C	M	T	M	W	G
Cluster24:1(1,0)	G	G	A	T	C	A	C	A	A	T	T	T	C	A	C	C	T	C	T	G
Cluster19:1(1,0)	A	A	R	T	C	G	C	A	R	Y	C	W	Y	M	Y	M	T	M	W	R
Cluster20:1(1,0)	A	R	R	W	C	R	Y	R	R	Y	T	T	C	A	C	M	W	M	W	R
Cluster9:1(1,0)	A	G	G	T	C	A	T	G	A	C	C	T	T	A	C	A	T	A	A	G
Cluster7:1(1,0)	A	A	G	T	C	G	T	G	A	T	T	T	C	C	T	A	T	C	T	A
Cluster5:1(1,0)	A	A	G	T	T	A	T	G	A	T	T	T	C	A	C	C	T	A	A	G
Cluster29:1(1,0)	A	A	G	W	T	A	T	G	R	C	C	T	T	A	C	A	T	A	A	G
Cluster15:1(1,0)	A	A	G	T	C	A	T	G	G	C	C	T	C	A	C	A	T	C	T	A
Cluster16:1(1,0)	G	G	G	T	C	A	T	G	G	T	T	T	T	A	C	A	A	C	T	A
Cluster31:1(1,0)	A	A	G	A	C	A	T	G	A	T	T	T	C	A	C	C	A	C	T	A
Cluster10:1(1,0)	A	A	G	A	T	A	T	G	G	C	C	T	T	A	C	A	T	C	T	A
Cluster18:1(1,0)	A	A	G	T	T	A	T	G	G	C	C	T	T	A	C	A	T	C	T	A
Cluster6:1(1,0)	A	A	G	A	T	A	T	G	G	C	T	T	C	A	C	A	T	C	T	A
Cluster25:1(1,0)	A	A	G	A	C	A	T	G	G	C	C	T	T	A	C	A	T	C	T	A
Cluster32:1(1,0)	A	A	G	A	T	A	T	G	G	T	T	T	T	A	Y	A	T	C	T	A
Cluster22:1(1,0)	A	A	G	A	C	A	T	G	G	C	C	T	T	A	C	A	T	C	T	A
Cluster21:1(1,0)	A	A	G	T	C	A	T	G	G	C	C	T	T	A	C	A	A	C	T	A

TS-011
TS-012
TS-121
TS-127
TS-135
TS-186
TS-296
TS-143
TS-296

MLO1	Solyc02g077570	SL2.50ch02	42467244	42472086

48.490.999; A>C; stop lost — Deleterious SNP

48.492.973; T>C; stop lost — Deleterious SNP

Haplotypes	0: 48489733	1: 48489923	2: 48490065	3: 48490453	4: 48490522	5: 48490568	6: 48490723	7: 48490768	8: 48490835	9: 48490993	10: 48490999	11: 48491010	12: 48491531	13: 48491909	14: 48491926	15: 48491953	16: 48492204	17: 48492445	18: 48492529	19: 48492609	20: 48492636	21: 48492782	22: 48492836	23: 48492973	24: 48493142	25: 48493477	26: 48493613	
Cluster16:1(1,0)	G	A	T	C	C	G	A	G	G	M	M	R	G	T	A	A	T	C	C	C	A	A	A	C	A	C	T	TS-214
Cluster21:1(1,0)	G	A	G	C	C	G	A	G	G	A	A	G	G	T	A	A	T	C	C	C	A	A	A	T	T	C	T	
Cluster12:1(1,0)	A	A	T	Y	G	G	A	R	G	A	A	G	R	Y	A	A	T	C	G	C	W	R	W	Y	W	C	T	TS-89
Cluster3:6(6,0)	A	A	T	C	G	G	A	A	G	A	A	G	G	T	A	A	T	C	G	C	T	G	T	T	T	C	T	
Cluster15:1(1,0)	A	A	G	Y	G	G	A	G	G	A	A	R	A	C	A	A	T	C	G	C	A	R	W	Y	A	C	A	
Cluster19:1(1,0)	A	A	T	C	S	R	A	A	G	A	A	G	G	T	A	A	T	C	G	C	T	G	T	T	T	C	T	
Cluster20:1(1,0)	A	A	G	C	G	G	A	A	G	A	A	G	G	T	A	A	T	C	G	C	T	G	W	T	W	C	T	
Cluster22:1(1,0)	A	A	G	C	G	G	A	A	G	A	A	G	G	T	A	A	T	C	G	C	T	R	T	Y	T	C	T	
Cluster5:2(2,0)	A	A	K	C	G	G	A	A	G	A	A	G	G	T	A	A	T	C	G	C	T	G	T	T	T	C	T	
Cluster9:1(1,0)	A	A	G	C	S	G	A	A	G	A	A	G	G	T	A	A	T	C	G	C	T	G	T	T	T	C	T	
Cluster14:1(1,0)	A	A	G	C	G	G	A	A	G	A	A	G	G	T	A	A	T	C	G	C	T	R	T	T	T	C	T	
Cluster0:140(140,0)	A	A	G	C	G	G	A	A	G	A	A	G	G	T	A	A	T	C	G	C	T	G	T	T	T	C	T	
Cluster4:2(2,0)	A	A	G	C	G	G	W	A	G	A	A	G	G	T	A	A	T	C	G	C	T	G	T	T	T	C	T	
Cluster7:1(1,0)	A	A	G	C	G	G	A	A	G	A	A	G	G	T	R	A	T	C	G	C	T	G	T	T	T	C	T	
Cluster11:1(1,0)	A	A	G	C	G	G	A	A	G	A	A	G	G	T	A	A	T	S	G	C	T	G	T	T	T	C	T	
Cluster2:6(6,0)	A	T	G	C	G	G	A	A	G	A	A	G	G	T	A	A	T	C	G	C	T	G	T	T	T	C	T	
Cluster6:2(2,0)	A	A	G	C	G	G	T	A	G	A	A	G	G	T	A	A	T	C	G	C	T	G	T	T	T	C	T	
Cluster8:1(1,0)	A	A	G	C	G	G	A	A	G	A	A	G	G	T	A	A	T	C	G	Y	T	G	T	T	T	Y	T	
Cluster13:1(1,0)	A	A	T	C	G	G	T	A	G	A	A	G	G	T	A	A	A	C	G	C	T	G	T	T	T	C	T	
Cluster23:1(1,0)	A	A	G	C	G	G	W	A	R	A	A	G	G	T	A	A	T	C	G	C	T	G	T	T	T	C	T	
Cluster10:1(1,0)	A	T	G	C	G	G	W	A	G	A	A	G	G	T	A	A	T	C	G	C	T	G	T	T	T	C	T	
Cluster1:10(10,0)	A	A	G	C	G	G	T	A	G	A	A	G	G	T	A	A	A	C	G	C	T	G	T	T	T	C	T	
Cluster18:1(1,0)	A	A	G	C	G	G	T	A	G	A	A	G	G	T	A	R	A	C	G	C	T	G	T	T	T	C	T	
Cluster17:1(1,0)	A	T	G	C	G	G	T	A	G	A	A	G	G	T	A	A	A	C	G	C	T	G	T	T	T	C	T	

**MLO1**	**Solyc06g082820**	**SL2.50ch06**	**48489673**	**48494035**

952.385; T>C; start lost

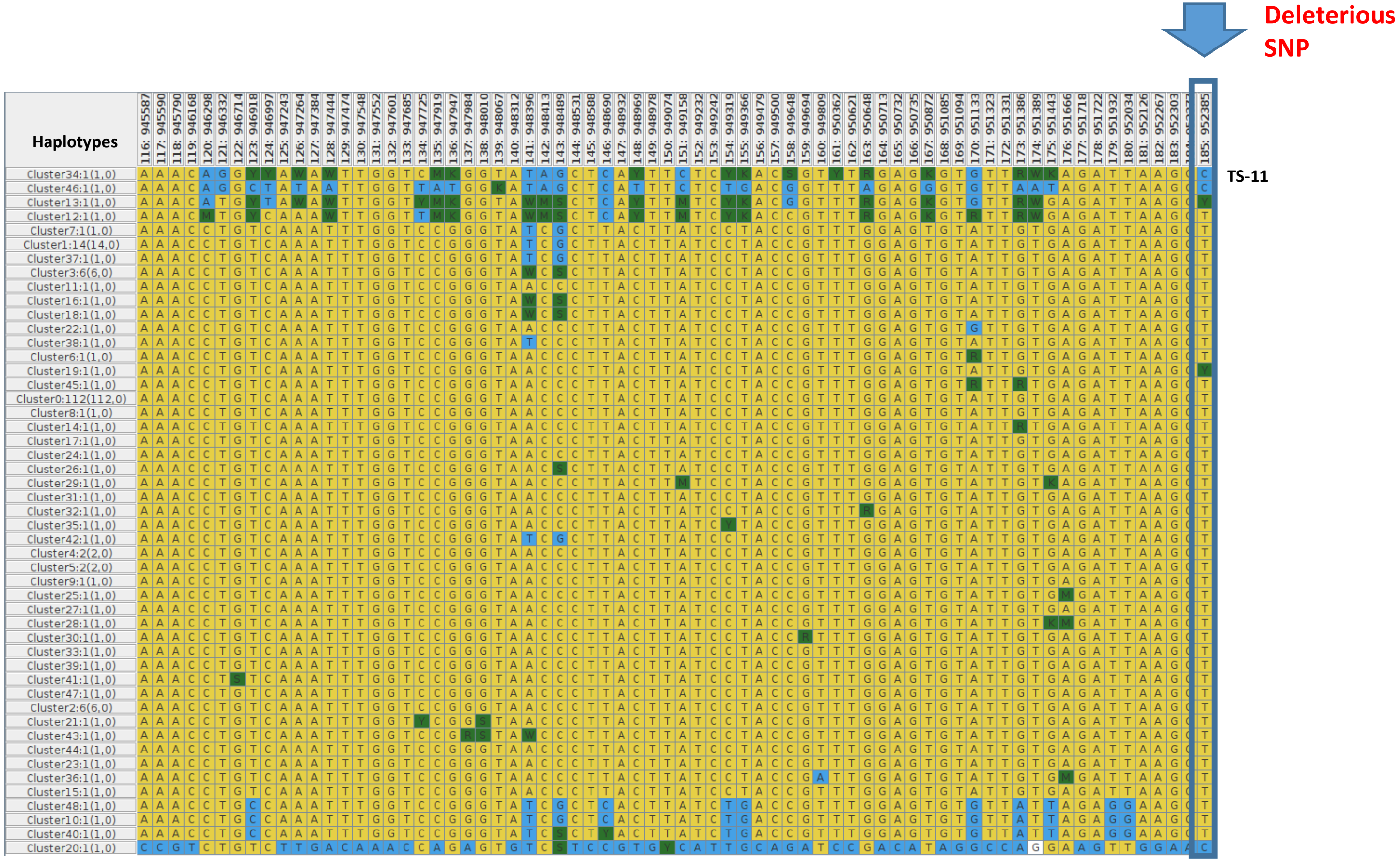

**PMR4**	**Solyc01g006350**	**SL2.50ch01**	**934429**	**952385**

81.135.674; C>T; stop gained

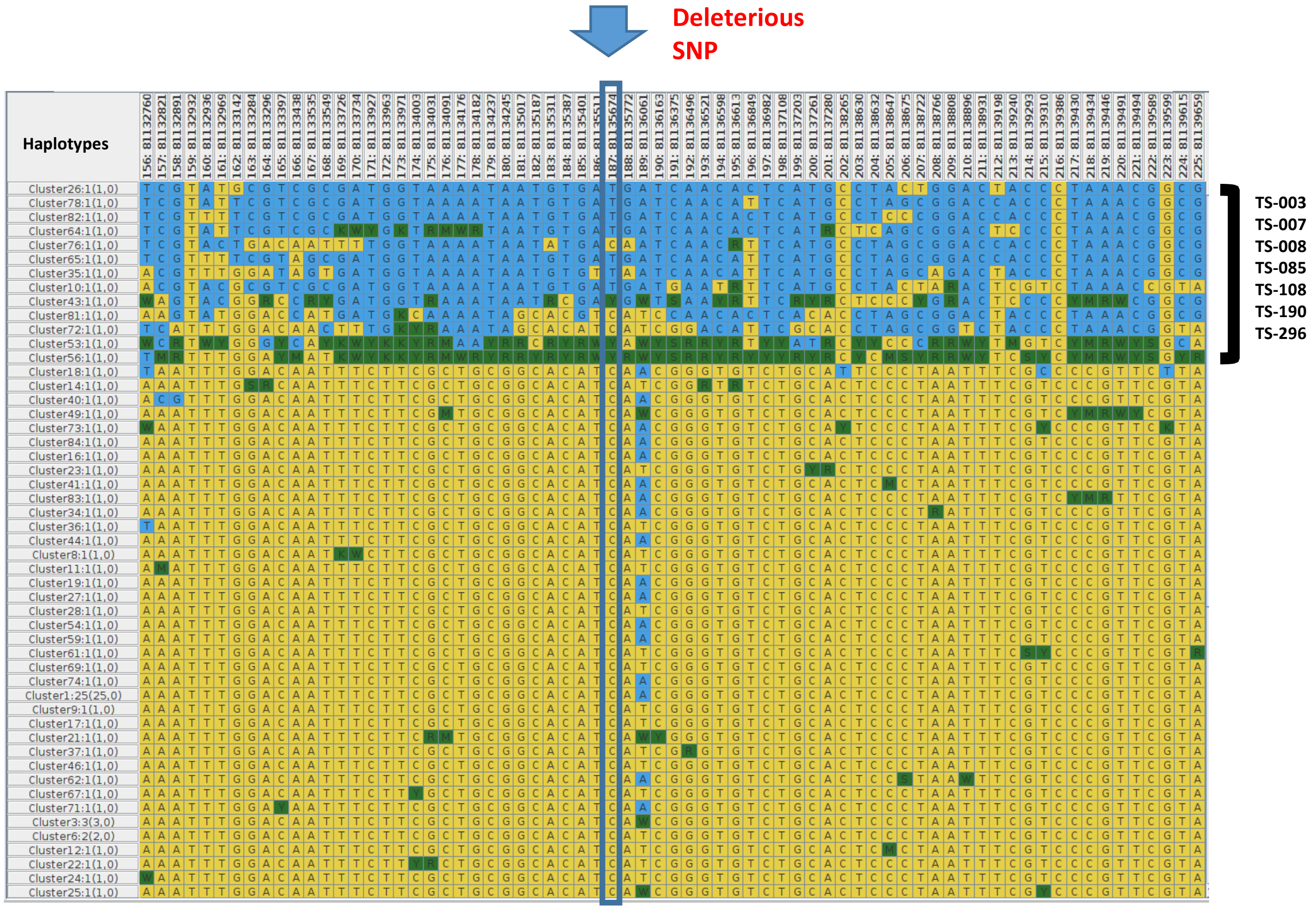

**PMR4**	**Solyc01g073750**	**SL2.50ch01**	**81118565**	**81146307**

64.110.648; A>C; stop lost

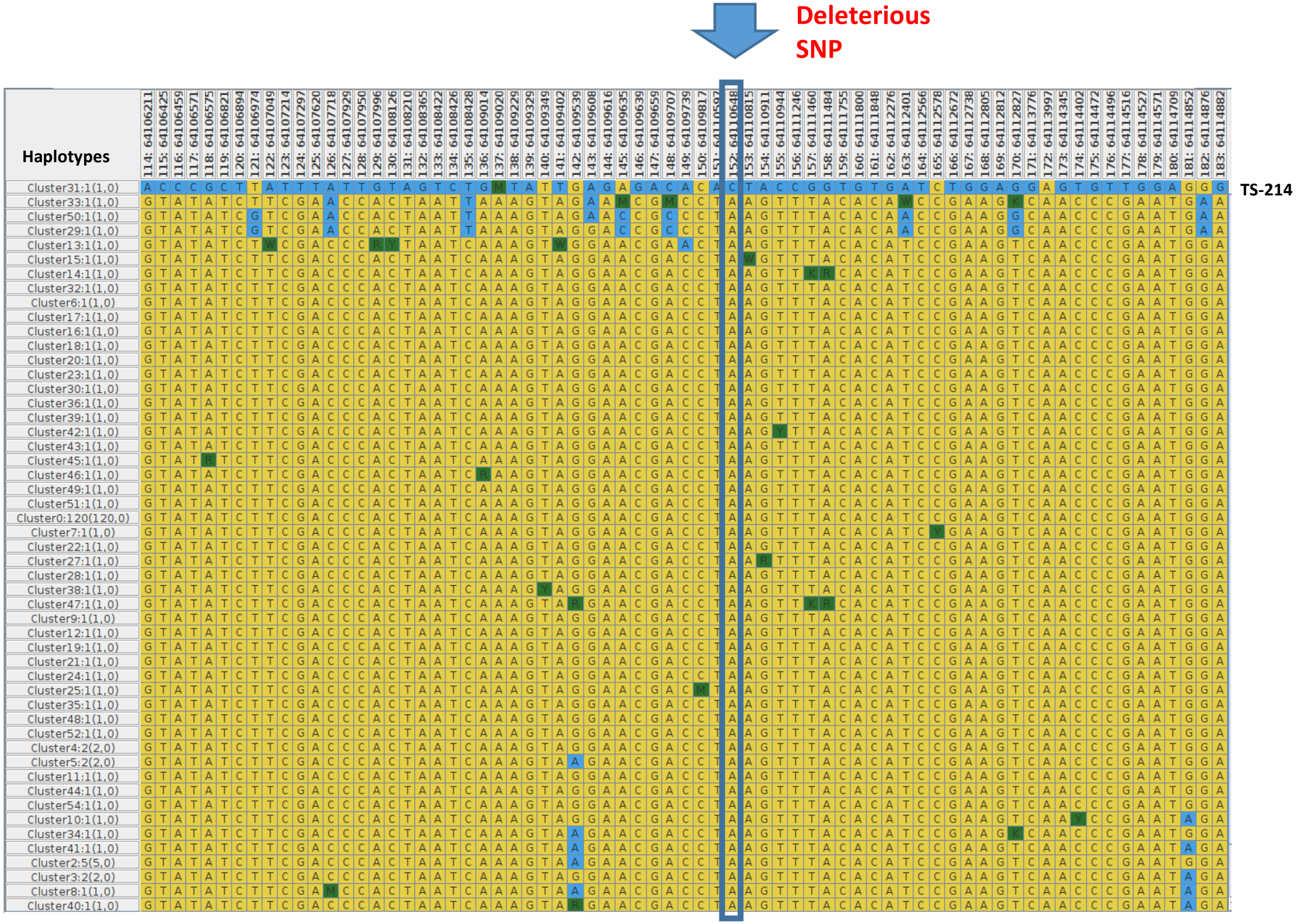

PMR4	Solyc07g056260	SL2.50ch07	64090068	64115779

46.562.565; A>T; stop lost

Deleterious SNP

Haplotypes	0: 46561755	1: 46561816	2: 46561936	3: 46561954	4: 46562075	5: 46562084	6: 46562154	7: 46562550	8: 46562565	9: 46562663	10: 46562716	11: 46562842	12: 46562867	13: 46562967	14: 46562971	15: 46563051	16: 46563098	17: 46563226	18: 46563251	19: 46563347	20: 46563410	21: 46563425	22: 46563456	23: 46563508	24: 46563511	25: 46563613	26: 46563731	
Cluster12:1(1,0)	K	Y	K	M	R	Y	K	W	W	W	R	A	A	K	T	Y	A	W	T	G	R	Y	M	C	Y	A	T	TS-68
Cluster0:171(171,0)	T	T	G	C	G	C	G	T	A	T	G	A	A	G	T	C	A	A	T	G	A	C	A	C	T	A	A	
Cluster2:1(1,0)	T	T	G	C	G	C	G	T	A	T	G	A	A	G	T	Y	A	A	T	G	A	C	A	C	T	A	A	
Cluster6:1(1,0)	T	T	G	C	G	C	G	T	A	T	G	A	A	G	T	C	A	W	T	G	A	C	A	C	T	A	A	
Cluster7:1(1,0)	T	T	G	C	G	C	G	T	A	T	G	A	A	G	T	C	A	A	T	G	R	Y	M	C	T	A	A	
Cluster11:1(1,0)	G	T	G	C	G	C	G	T	A	T	G	A	A	G	T	C	A	A	T	G	A	C	A	C	T	A	A	
Cluster4:1(1,0)	T	T	G	C	G	C	G	T	A	T	G	A	A	G	T	C	A	A	T	G	A	C	A	Y	Y	A	A	
Cluster9:1(1,0)	T	T	G	C	G	C	G	T	A	T	G	A	R	G	T	C	A	A	T	G	A	C	A	C	T	A	A	
Cluster3:1(1,0)	K	T	G	C	G	C	G	T	A	T	G	W	R	G	T	C	A	A	T	G	A	C	A	C	T	A	A	
Cluster13:1(1,0)	G	T	G	C	G	C	G	T	A	T	G	A	R	T	A	T	A	A	T	C	G	C	A	C	T	A	A	
Cluster1:2(2,0)	K	T	G	C	G	C	G	T	A	T	G	W	R	K	W	Y	M	A	Y	G	R	C	A	C	T	R	A	
Cluster5:1(1,0)	T	T	G	C	G	C	G	T	A	T	G	T	G	T	A	T	C	A	T	G	G	C	A	C	T	A	A	
Cluster10:1(1,0)	T	T	G	C	G	C	G	T	A	T	G	T	R	T	A	T	C	A	C	G	G	C	A	C	T	G	A	
Cluster8:1(1,0)	G	T	G	C	G	C	G	T	A	T	G	T	G	T	A	T	C	A	C	G	G	C	A	C	T	G	A	

PMR5	Solyc02g082950	SL2.50ch02	46561600	46563900

852.558; T>A; stop lost

Deleterious SNP

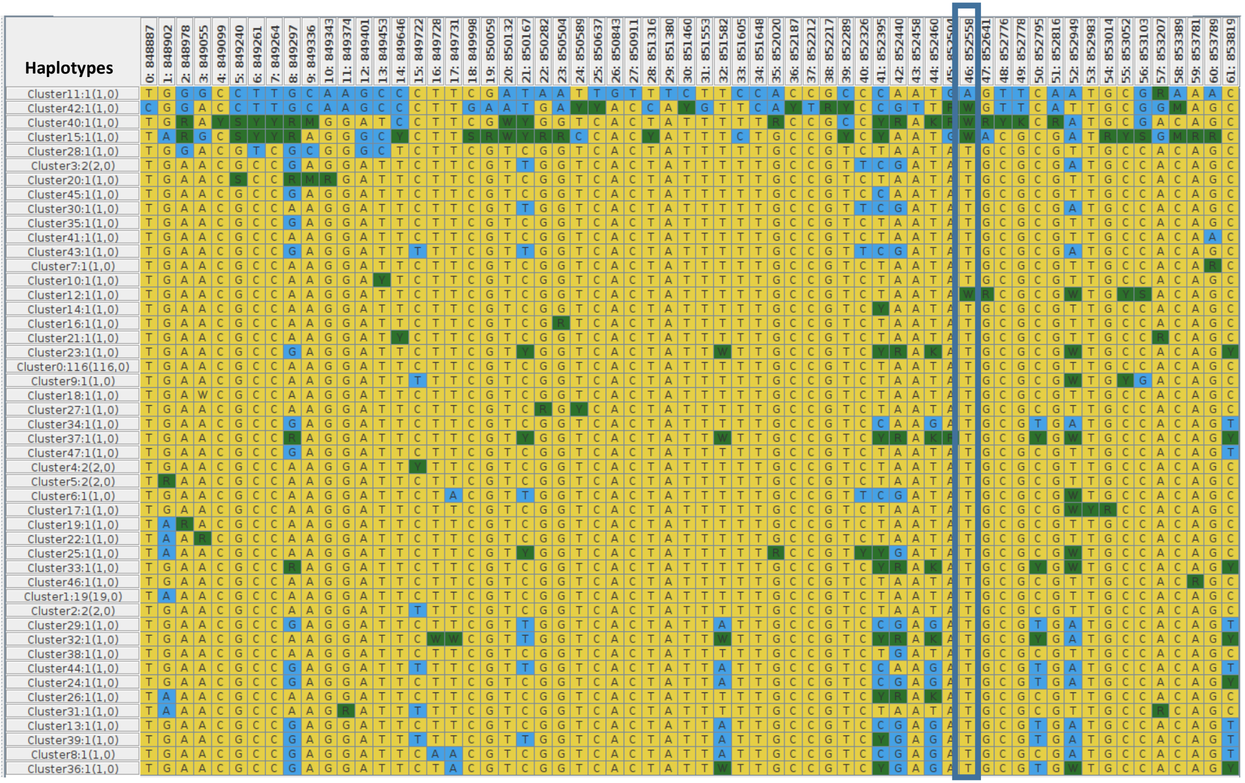

TS-127
TS-409

**PMR5**	**Solyc03g006220**	**SL2.50ch03**	**848503**	**853887**

61.809.964; A>G; stop lost

Deleterious SNP

TS-143

Haplotypes	0: 61806397	1: 61806401	2: 61806403	3: 61806422	4: 61806499	5: 61806524	6: 61806625	7: 61806646	8: 61806738	9: 61806748	10: 61806998	11: 61807000	12: 61807198	13: 61807419	14: 61807464	15: 61808404	16: 61808495	17: 61808524	18: 61808577	19: 61808638	20: 61808701	21: 61808710	22: 61808745	23: 61808789	24: 61808861	25: 61808863	26: 61809042	27: 61809107	28: 61809109	29: 61809252
Cluster13:1(1,0)	T	C	G	G	C	C	C	C	G	A	T	C	A	A	T	T	A	C	G	T	T	A	A	T	G	T	C	G	A	C
Cluster0:123(123,0)	T	C	G	G	T	C	C	C	G	A	T	C	A	A	T	T	A	C	G	T	T	A	A	T	G	T	C	G	A	C
Cluster15:1(1,0)	T	C	G	G	Y	Y	C	C	G	A	T	C	A	A	T	T	A	C	G	T	T	A	A	T	G	T	C	G	A	C
Cluster1:14(14,0)	T	C	G	G	T	C	C	C	G	A	T	C	A	A	T	T	A	C	G	T	T	A	A	T	G	T	C	G	R	C
Cluster2:10(10,0)	T	C	G	G	T	C	C	C	G	A	T	C	A	A	T	T	A	C	G	T	T	A	A	T	G	T	C	G	A	C
Cluster4:4(4,0)	T	C	G	G	T	C	C	C	G	A	T	C	A	A	T	T	A	C	G	T	T	A	A	T	G	T	C	R	A	C
Cluster8:1(1,0)	T	C	G	G	T	C	C	C	G	A	T	C	A	A	T	T	A	C	G	T	T	A	A	T	G	T	C	G	A	C
Cluster16:1(1,0)	T	C	G	G	T	C	C	C	G	A	T	C	A	A	T	T	A	Y	G	T	T	A	A	T	G	T	C	G	A	C
Cluster18:1(1,0)	T	C	G	G	T	C	C	C	G	A	T	C	A	A	T	T	A	C	G	T	T	A	A	T	G	T	C	G	A	C
Cluster3:5(5,0)	T	C	G	G	T	C	C	C	G	A	T	C	A	A	T	T	A	C	G	T	T	A	A	T	G	T	C	G	A	C
Cluster5:4(4,0)	T	C	G	G	T	C	C	C	G	A	T	C	A	A	T	T	A	C	G	T	T	A	A	T	G	T	C	R	R	C
Cluster6:4(4,0)	T	C	G	G	T	C	C	C	G	A	T	C	T	A	T	T	A	C	G	T	T	A	A	T	G	T	C	G	A	C
Cluster7:2(2,0)	T	C	G	G	T	C	C	C	G	A	T	C	A	A	T	T	A	C	G	T	T	A	A	T	G	T	C	A	A	C
Cluster11:1(1,0)	T	C	G	G	T	C	C	C	G	A	T	C	A	A	T	T	A	C	G	T	T	A	A	T	G	T	C	G	G	C
Cluster19:1(1,0)	T	C	G	G	T	C	C	C	G	A	T	C	A	A	T	T	A	C	G	T	T	A	W	Y	G	T	C	G	A	C
Cluster20:1(1,0)	T	Y	G	G	T	C	C	C	G	R	T	C	A	A	T	T	A	C	G	T	T	A	A	T	G	T	C	G	A	C
Cluster23:1(1,0)	T	C	G	G	T	C	C	C	G	A	T	S	A	A	T	T	A	C	G	T	T	A	A	T	G	T	C	G	A	C
Cluster17:1(1,0)	T	C	G	G	T	C	C	C	G	A	T	C	A	A	T	T	A	C	G	T	T	A	A	T	G	T	C	G	A	C
Cluster21:1(1,0)	T	Y	G	G	T	C	C	C	G	R	T	C	A	A	T	T	A	C	G	T	T	A	A	T	G	T	C	G	A	C
Cluster10:1(1,0)	Y	C	K	G	T	C	C	S	G	A	T	C	A	A	T	T	A	C	G	T	T	A	A	T	G	T	C	G	A	C
Cluster22:1(1,0)	T	C	G	T	T	C	C	C	G	A	T	G	A	A	T	T	A	C	G	T	T	A	A	T	G	T	C	G	G	C
Cluster24:1(1,0)	T	C	G	G	T	C	C	C	G	A	T	G	A	A	T	T	A	C	A	T	T	A	A	T	G	T	C	G	A	C
Cluster25:1(1,0)	T	Y	G	G	Y	Y	C	C	G	A	T	C	A	A	T	T	W	C	R	C	Y	R	W	Y	G	Y	C	G	A	C
Cluster12:1(1,0)	C	C	T	G	T	Y	S	C	R	R	T	S	A	A	A	T	A	C	G	Y	C	G	A	T	G	T	C	G	A	C
Cluster14:1(1,0)	C	C	T	G	C	T	C	C	A	G	T	C	A	A	T	G	A	C	G	C	C	G	T	C	G	T	G	A	A	C
Cluster26:1(1,0)	T	Y	G	G	C	Y	C	C	G	R	T	S	A	A	A	T	A	C	G	Y	C	G	T	C	R	T	C	R	A	C
Cluster9:1(1,0)	T	T	G	G	C	T	C	C	G	G	C	G	A	T	A	T	T	C	A	C	C	G	T	C	G	C	C	G	R	A

Haplotypes	30: 61809366	31: 61809416	32: 61809544	33: 61809654	34: 61809787	35: 61809790	36: 61809804	37: 61809828	38: 61809842	39: 61809861	40: 61809949	41: 61809964	42: 61810484	43: 61810555	44: 61810638	45: 61810722	46: 61810752	47: 61810812	48: 61810946	49: 61811048	50: 61811280	51: 61811284	52: 61811343	53: 61811353	54: 61811377	55: 61811401	56: 61811434	57: 61811486	58: 61811492
Cluster13:1(1,0)	A	A	A	C	G	C	T	T	G	G	C	G	C	G	A	A	A	T	G	G	C	A	T	G	C	A	G	C	A
Cluster0:123(123,0)	A	A	A	C	G	C	T	T	G	G	C	A	C	G	A	A	A	T	G	G	C	A	T	G	C	A	G	C	A
Cluster15:1(1,0)	A	A	A	C	G	C	T	T	G	G	C	A	C	G	A	A	A	T	G	G	C	A	T	G	C	A	G	C	A
Cluster1:14(14,0)	A	A	A	C	G	C	T	T	G	G	C	A	C	G	A	A	A	T	G	G	C	A	T	G	C	A	G	C	A
Cluster2:10(10,0)	A	A	A	C	G	C	T	T	G	G	C	A	C	G	A	A	A	T	K	G	C	A	T	G	C	A	G	C	A
Cluster4:4(4,0)	A	A	A	C	G	C	T	T	G	G	C	A	C	G	A	A	A	T	G	G	C	A	T	G	C	A	G	C	A
Cluster8:1(1,0)	A	A	A	C	G	C	T	T	G	G	C	A	C	G	A	A	A	T	G	G	C	A	T	R	C	A	G	C	A
Cluster16:1(1,0)	A	A	A	C	G	C	T	T	G	G	C	A	C	G	A	A	A	T	G	G	C	A	T	G	C	A	G	C	A
Cluster18:1(1,0)	A	A	A	C	G	C	T	T	G	G	C	A	C	R	A	A	A	T	G	G	C	A	T	G	C	A	G	C	A
Cluster3:5(5,0)	A	A	A	C	G	C	T	T	G	G	C	A	C	G	A	A	A	T	T	G	C	A	T	G	C	A	G	C	A
Cluster5:4(4,0)	A	A	A	C	G	C	T	T	G	G	C	A	C	G	A	A	A	T	G	G	C	A	T	G	C	A	G	C	A
Cluster6:4(4,0)	A	A	A	C	G	C	T	T	G	G	C	A	C	G	A	A	A	T	G	G	C	A	T	G	C	A	G	C	A
Cluster7:2(2,0)	A	A	A	C	G	C	T	T	G	G	C	A	C	G	A	A	A	T	G	G	C	A	T	G	C	A	G	C	A
Cluster11:1(1,0)	A	A	A	C	G	C	T	T	G	G	C	A	C	G	A	A	A	T	G	G	C	A	T	G	C	A	G	C	A
Cluster19:1(1,0)	A	A	A	C	G	C	T	T	G	G	C	A	C	G	A	A	A	T	G	G	C	A	T	G	C	A	G	C	A
Cluster20:1(1,0)	A	A	A	C	G	C	T	T	G	G	C	A	C	G	A	A	A	T	G	G	C	A	T	G	C	A	G	C	A
Cluster23:1(1,0)	A	A	A	C	G	C	T	T	G	G	C	A	C	G	A	A	A	T	K	G	C	A	T	G	C	A	G	C	A
Cluster17:1(1,0)	A	A	A	C	G	C	T	T	G	G	C	A	C	G	A	A	A	T	K	G	C	A	T	G	C	A	G	S	W
Cluster21:1(1,0)	A	A	A	C	G	C	T	T	G	G	C	A	C	G	A	A	A	T	K	G	C	A	T	G	C	A	G	C	A
Cluster10:1(1,0)	A	A	A	C	G	C	T	T	G	G	C	A	C	G	A	A	A	T	T	G	C	A	T	G	C	A	G	C	A
Cluster22:1(1,0)	A	A	A	C	G	C	T	T	G	G	C	A	C	G	A	A	A	T	G	G	C	A	T	G	C	A	G	C	A
Cluster24:1(1,0)	A	A	A	C	G	C	T	T	G	A	C	A	C	G	A	A	A	T	G	G	C	A	T	G	C	A	G	C	A
Cluster25:1(1,0)	A	A	A	C	G	C	T	T	G	G	C	A	C	G	A	A	A	T	G	G	C	A	T	G	C	A	G	C	A
Cluster12:1(1,0)	G	A	R	Y	G	C	T	Y	S	G	Y	A	Y	G	A	R	R	T	K	S	Y	W	Y	G	Y	W	K	S	W
Cluster14:1(1,0)	A	A	A	C	G	A	C	C	C	G	T	A	T	G	A	A	A	T	G	G	C	A	T	G	C	A	G	C	A
Cluster26:1(1,0)	A	A	R	T	G	C	Y	Y	S	G	C	A	C	A	G	G	G	C	T	G	C	A	C	G	C	A	G	S	W
Cluster9:1(1,0)	A	T	A	T	A	C	T	C	C	A	C	A	C	A	G	G	G	C	T	G	C	A	C	G	C	A	G	G	T

PMR5 Solyc07g053350 SL2.50ch07 61806270 61811621

37.648.327; G>A; Intron variant

Deleterious SNP

TS-186

Haplotypes	0: 37643831	1: 37643917	2: 37644015	3: 37644231	4: 37644396	5: 37644599	6: 37644937	7: 37645152	8: 37645173	9: 37645185	10: 37645223	11: 37645564	12: 37645683	13: 37645694	14: 37645714	15: 37646077	16: 37646085	17: 37646234	18: 37646527	19: 37646531	20: 37646711	21: 37646778
Cluster29:1(1,0)	T	G	C	T	A	G	T	G	G	A	T	T	A	A	G	T	C	G	G	T	T	C
Cluster0:64(64,0)	T	G	C	T	A	G	G	A	A	G	C	T	A	A	G	T	C	G	G	T	T	C
Cluster28:1(1,0)	T	K	C	T	A	G	G	A	A	G	C	T	A	A	G	T	C	G	G	T	T	C
Cluster3:8(8,0)	T	G	C	T	A	G	G	A	A	G	C	T	A	A	G	C	C	G	G	T	T	C
Cluster8:2(2,0)	T	G	C	T	A	G	G	A	A	G	C	T	A	A	G	T	C	G	G	T	T	C
Cluster9:2(2,0)	T	G	C	T	A	G	G	A	A	G	C	T	A	A	G	Y	Y	G	G	T	T	C
Cluster25:1(1,0)	T	G	C	T	T	G	G	A	A	G	C	T	A	A	G	T	C	G	G	T	T	C
Cluster22:1(1,0)	T	G	C	T	A	G	G	A	A	G	C	T	A	A	G	Y	T	G	G	T	T	C
Cluster23:1(1,0)	T	G	C	T	A	G	G	A	A	G	C	T	A	A	G	C	C	G	G	T	T	C
Cluster1:54(54,0)	T	G	C	T	A	G	G	A	A	G	C	T	A	A	G	C	T	G	G	T	T	C
Cluster10:2(2,0)	T	G	C	T	A	G	G	A	A	G	C	T	A	A	G	C	C	G	G	T	T	C
Cluster17:1(1,0)	T	G	C	T	A	G	G	A	A	G	C	T	A	A	G	C	C	G	G	T	T	C
Cluster32:1(1,0)	T	T	C	T	A	G	G	A	A	G	C	T	A	G	G	T	C	G	G	T	T	C
Cluster35:1(1,0)	T	G	C	T	A	G	G	A	A	G	C	T	A	A	G	T	C	G	G	T	T	A
Cluster39:1(1,0)	T	G	C	T	A	G	G	A	A	G	C	T	A	A	G	T	C	G	G	T	T	A
Cluster6:2(2,0)	T	G	C	T	A	G	G	A	A	G	C	T	A	A	G	C	T	G	G	K	T	C
Cluster15:1(1,0)	T	G	C	T	A	G	G	A	A	G	C	Y	A	A	G	C	T	G	G	T	T	C
Cluster24:1(1,0)	T	G	C	T	A	G	G	A	A	G	C	T	A	A	G	C	T	G	G	T	T	C
Cluster27:1(1,0)	T	G	C	T	A	G	G	A	A	G	C	T	A	A	G	C	T	G	G	T	T	C
Cluster30:1(1,0)	T	G	C	T	A	G	G	A	A	G	C	T	A	A	G	T	C	G	G	T	T	M
Cluster31:1(1,0)	T	G	C	T	A	G	G	A	A	G	C	T	A	R	G	C	T	G	G	T	T	C
Cluster36:1(1,0)	T	G	C	T	A	G	G	A	A	G	C	T	A	A	G	C	C	G	G	T	T	M
Cluster2:8(8,0)	T	G	C	T	A	G	G	A	A	G	C	T	A	A	G	C	T	G	G	T	T	C
Cluster5:4(4,0)	T	G	C	T	A	G	G	A	A	G	C	T	A	A	G	T	C	G	G	T	T	A
Cluster7:2(2,0)	T	G	C	T	A	G	G	A	A	G	C	T	A	A	G	C	C	G	G	T	T	A
Cluster11:2(2,0)	T	G	C	T	A	G	G	A	A	G	C	T	A	A	G	C	C	G	G	T	T	C
Cluster13:1(1,0)	T	G	C	T	A	G	G	A	A	G	C	T	A	A	G	C	Y	G	G	T	T	C
Cluster16:1(1,0)	T	G	C	Y	A	R	G	A	A	G	C	T	A	A	G	C	T	G	G	T	T	C
Cluster26:1(1,0)	T	G	C	T	A	G	G	A	A	G	C	T	A	A	G	C	Y	G	G	T	T	M
Cluster18:1(1,0)	Y	G	C	Y	A	G	G	A	A	G	C	T	A	A	G	C	C	G	G	T	T	M
Cluster21:1(1,0)	T	G	C	T	A	G	G	A	A	G	C	T	A	A	G	C	Y	G	G	T	T	M
Cluster34:1(1,0)	T	G	C	T	A	G	G	A	A	G	C	T	A	A	G	C	T	G	G	T	T	C
Cluster40:1(1,0)	T	G	C	T	A	G	G	A	A	G	C	T	A	A	G	C	C	G	G	T	T	A
Cluster4:6(6,0)	T	G	C	T	A	G	G	A	A	G	C	T	A	A	G	C	C	G	G	T	T	A
Cluster12:1(1,0)	T	G	C	T	A	G	G	A	A	G	C	T	A	A	G	C	C	G	G	T	T	A
Cluster33:1(1,0)	T	G	C	T	A	G	G	A	A	G	C	T	A	A	G	C	C	G	G	K	T	A
Cluster19:1(1,0)	T	G	C	T	A	G	G	A	A	G	C	T	A	A	G	C	C	G	S	G	T	A
Cluster37:1(1,0)	T	G	C	T	T	G	G	A	A	G	C	T	A	A	A	T	C	K	G	T	W	A
Cluster20:1(1,0)	T	G	C	T	A	G	G	A	A	G	C	T	G	A	G	T	C	T	G	T	A	A
Cluster38:1(1,0)	T	G	C	T	A	G	G	A	A	G	C	T	G	A	G	T	C	T	G	T	A	A
Cluster14:1(1,0)	T	G	A	T	A	G	G	A	A	G	C	T	A	A	A	T	C	T	G	T	A	A

Haplotypes	22: 37646852	23: 37646855	24: 37647017	25: 37647036	26: 37647213	27: 37647413	28: 37647452	29: 37647609	30: 37647694	31: 37647757	32: 37647937	33: 37647951	34: 37648069	35: 37648228	36: 37648288	37: 37648316	38: 37648327	39: 37648399	40: 37648472	41: 37648668	42: 37648956
Cluster29:1(1,0)	T	C	C	T	C	A	T	G	G	A	A	G	A	G	A	A	A	G	T	C	A
Cluster0:64(64,0)	T	C	C	T	C	A	T	G	T	A	A	C	G	G	G	T	G	A	T	C	A
Cluster28:1(1,0)	T	C	C	T	C	A	T	G	T	A	A	C	G	G	G	T	G	A	T	C	A
Cluster3:8(8,0)	T	C	C	T	C	A	T	G	T	A	A	C	G	G	G	T	G	A	T	C	A
Cluster8:2(2,0)	T	T	C	T	C	A	T	G	T	A	A	C	G	G	G	T	G	A	T	C	A
Cluster9:2(2,0)	T	C	C	T	C	A	T	G	T	A	A	C	G	G	G	T	G	A	T	C	A
Cluster25:1(1,0)	T	C	C	T	C	A	T	G	T	A	A	C	G	G	G	T	G	A	T	C	A
Cluster22:1(1,0)	T	C	C	T	C	A	T	G	T	A	A	C	G	G	G	T	G	A	T	C	A
Cluster23:1(1,0)	T	C	C	T	C	A	T	G	T	R	A	C	G	G	G	T	G	A	T	C	A
Cluster1:54(54,0)	T	C	C	T	C	A	T	G	T	A	A	C	G	G	G	T	G	A	T	C	A
Cluster10:2(2,0)	T	C	C	T	C	T	T	G	T	A	A	C	G	G	G	T	G	A	T	C	A
Cluster17:1(1,0)	T	C	C	W	C	A	T	G	T	R	A	C	G	G	G	T	G	A	T	C	A
Cluster32:1(1,0)	T	C	G	T	C	A	T	G	T	A	A	C	G	G	G	A	G	A	T	C	A
Cluster35:1(1,0)	T	C	C	T	C	A	T	G	T	G	A	C	G	G	G	T	G	A	T	C	A
Cluster39:1(1,0)	T	C	C	A	C	A	T	G	T	A	A	C	G	G	G	T	G	A	T	C	A
Cluster6:2(2,0)	T	C	C	T	C	A	T	G	T	A	A	C	G	G	G	T	G	A	T	C	A
Cluster15:1(1,0)	T	C	C	T	C	A	T	G	T	A	A	C	G	G	G	T	G	A	T	C	A
Cluster24:1(1,0)	T	C	C	T	C	A	T	G	T	A	A	C	G	R	G	T	G	A	T	C	A
Cluster27:1(1,0)	T	C	C	T	C	A	T	G	T	A	A	C	G	G	G	T	G	A	Y	C	A
Cluster30:1(1,0)	T	C	C	A	C	A	T	G	T	G	A	C	G	G	G	T	G	A	T	C	A
Cluster31:1(1,0)	T	C	C	T	C	A	T	G	T	A	A	C	G	G	G	T	G	A	T	C	A
Cluster36:1(1,0)	T	C	C	W	C	A	T	G	T	R	A	C	G	G	G	T	G	A	T	C	A
Cluster2:8(8,0)	T	T	C	T	C	A	T	G	T	A	A	C	G	G	G	T	G	A	T	C	A
Cluster5:4(4,0)	T	C	C	A	C	A	T	G	T	G	A	C	G	G	G	T	G	A	T	C	A
Cluster7:2(2,0)	T	C	C	T	C	A	T	G	T	G	A	C	G	G	G	T	G	A	T	C	A
Cluster11:2(2,0)	T	C	C	A	C	A	T	G	T	G	A	C	G	G	G	T	G	A	T	C	A
Cluster13:1(1,0)	T	C	C	A	C	A	T	G	T	R	A	C	G	G	G	T	G	A	T	C	A
Cluster16:1(1,0)	T	C	C	T	C	A	T	G	T	A	A	C	G	G	G	T	G	A	T	C	A
Cluster26:1(1,0)	T	C	C	W	C	A	T	G	T	R	A	C	G	G	G	T	G	A	T	C	A
Cluster18:1(1,0)	T	C	C	W	C	A	T	G	T	R	A	C	G	G	G	T	G	A	T	C	A
Cluster21:1(1,0)	T	C	C	A	C	A	T	G	T	R	A	C	G	G	G	T	G	A	T	C	A
Cluster34:1(1,0)	T	T	C	W	C	A	T	G	T	A	A	C	G	G	G	T	G	A	T	C	A
Cluster40:1(1,0)	T	C	C	W	C	A	T	G	T	G	A	C	G	G	G	T	G	A	T	C	A
Cluster4:6(6,0)	T	C	C	A	C	A	T	G	T	G	A	C	G	G	G	T	G	A	T	C	A
Cluster12:1(1,0)	T	C	C	A	C	A	T	G	T	G	A	C	G	G	G	T	G	A	T	Y	A
Cluster33:1(1,0)	T	C	C	A	C	A	T	G	T	G	A	C	G	G	G	T	G	A	T	C	A
Cluster19:1(1,0)	T	C	C	A	C	A	T	G	T	G	A	C	G	G	G	T	G	A	T	C	A
Cluster37:1(1,0)	C	C	C	T	C	A	T	A	T	A	A	C	G	G	G	A	G	A	T	C	T
Cluster20:1(1,0)	C	C	C	T	T	A	T	A	T	A	G	C	G	G	G	A	G	A	C	C	A
Cluster38:1(1,0)	C	C	C	T	C	A	A	A	T	A	G	C	G	G	G	A	G	A	C	C	A
Cluster14:1(1,0)	C	C	C	T	C	A	A	G	T	A	G	C	G	G	G	A	G	A	C	C	T

PMR6	Solyc02g067450	SL2.50ch02	37643757	37649399

45.011.463; G>T; stop gained

Deleterious SNP

Haplotypes	0: 45010472	1: 45010703	2: 45010722	3: 45010743	4: 45010774	5: 45010864	6: 45010873	7: 45011400	8: 45011463	9: 45011508	10: 45011523	11: 45011697	12: 45011745	13: 45011763	14: 45011784	15: 45011799	16: 45011849	17: 45011889	18: 45012199	19: 45012207	20: 45012222	21: 45012579	22: 45012880	23: 45012884	24: 45012886	25: 45013236	26: 45013763	27: 45013805	28: 45013810	29: 45014506	30: 45014744	31: 45015163	32: 45015181	33: 45015212	34: 45015448	35: 45015475
Cluster0:91(91,0)	T	A	G	G	G	A	A	A	G	C	G	A	C	G	G	T	G	A	G	A	T	C	A	A	A	C	C	C	T	T	A	A	G	C	G	G
Cluster8:1(1,0)	T	A	G	G	G	M	R	A	G	C	G	A	C	G	G	T	G	A	G	A	T	C	A	A	A	C	C	C	T	T	A	A	G	C	G	G
Cluster25:1(1,0)	T	A	G	G	G	A	R	A	G	C	G	A	C	G	G	T	G	A	G	A	T	C	A	A	A	C	C	C	T	T	A	A	G	C	G	G
Cluster2:8(8,0)	T	A	G	G	G	A	A	A	G	C	G	A	C	G	G	T	G	A	G	A	T	C	A	A	A	C	C	C	T	T	A	R	G	C	G	G
Cluster6:1(1,0)	T	A	G	K	G	M	A	A	G	C	G	A	C	G	G	T	G	A	G	A	T	C	A	A	A	C	C	C	T	T	A	A	G	C	G	G
Cluster18:1(1,0)	T	A	G	G	G	A	A	A	G	C	G	A	C	G	G	T	G	A	G	A	T	C	A	A	A	C	C	C	T	T	A	A	G	C	R	G
Cluster21:1(1,0)	T	R	G	G	G	A	A	A	G	C	G	A	C	G	G	T	G	A	G	A	T	C	A	A	A	C	C	C	T	T	A	A	G	C	G	G
Cluster24:1(1,0)	T	A	G	G	G	A	A	A	G	C	G	A	C	G	G	T	G	A	G	A	T	C	A	A	A	C	Y	C	T	T	A	A	G	C	G	G
Cluster28:1(1,0)	T	A	G	G	G	A	A	A	G	C	G	A	C	G	G	T	G	A	R	A	T	C	A	A	A	C	C	C	T	T	A	A	G	C	G	G
Cluster29:1(1,0)	T	A	G	G	G	A	A	R	G	C	G	A	C	G	G	T	G	A	G	A	T	C	A	A	A	C	C	C	T	T	A	A	G	C	G	G
Cluster1:52(52,0)	T	A	G	G	G	A	A	A	G	C	G	A	C	G	G	T	G	A	G	A	T	C	A	A	A	C	C	C	T	T	A	G	G	C	G	G
Cluster10:1(1,0)	T	A	G	G	G	A	A	A	G	C	G	A	C	G	G	T	G	A	G	A	T	C	A	A	A	C	C	C	T	T	A	R	R	C	G	G
Cluster12:1(1,0)	T	A	G	G	G	A	A	A	G	Y	K	A	C	G	G	T	G	A	G	A	T	C	A	A	A	C	C	C	T	T	A	A	G	C	G	G
Cluster14:1(1,0)	T	A	G	G	G	A	A	A	G	C	G	A	C	R	G	T	G	A	G	A	T	C	A	A	A	C	C	C	T	T	A	R	G	C	G	G
Cluster3:4(4,0)	T	A	G	G	G	A	A	A	G	C	G	A	C	G	G	T	G	A	G	W	T	C	A	A	A	C	C	C	T	T	A	G	G	C	G	G
Cluster4:4(4,0)	T	A	G	G	G	A	A	A	G	C	G	A	C	G	G	T	G	A	G	A	W	C	A	A	A	C	C	C	T	T	A	G	G	C	G	G
Cluster7:1(1,0)	T	A	G	G	G	A	A	A	K	C	G	A	C	G	G	T	G	A	G	A	T	C	A	A	A	C	C	C	T	T	A	G	G	C	G	G
Cluster9:1(1,0)	T	A	G	G	G	A	A	A	G	C	G	A	C	G	G	T	G	A	G	A	T	C	A	W	A	C	C	C	T	T	A	G	G	C	G	G
Cluster19:1(1,0)	T	A	G	G	G	A	A	A	G	Y	G	A	C	G	G	T	G	A	G	A	T	C	A	A	A	C	C	C	T	T	A	G	G	C	G	G
Cluster20:1(1,0)	T	A	G	G	G	A	A	A	G	C	G	A	C	G	G	T	G	A	G	A	T	Y	A	A	A	C	C	C	T	T	A	G	G	C	G	G
Cluster22:1(1,0)	T	A	G	K	G	A	A	A	G	C	G	A	C	G	G	T	G	A	G	A	T	C	A	A	A	C	C	C	T	T	A	G	G	C	G	G
Cluster5:2(2,0)	T	A	G	G	G	A	A	A	G	C	G	A	C	G	G	T	G	A	G	A	A	C	A	A	A	C	C	C	T	T	A	G	G	C	G	G
Cluster16:1(1,0)	T	A	G	G	G	A	A	A	G	C	G	A	C	G	G	T	G	A	G	W	W	C	A	A	A	C	C	C	T	T	A	G	G	C	G	G
Cluster23:1(1,0)	T	A	G	G	G	A	A	A	G	C	G	A	C	G	G	T	G	A	G	T	T	C	A	A	A	C	C	C	T	T	A	G	G	C	G	G
Cluster15:1(1,0)	T	A	G	G	G	A	A	A	G	Y	G	A	C	G	G	T	S	W	G	A	T	C	A	A	A	C	C	C	T	T	A	G	G	C	G	G
Cluster17:1(1,0)	T	R	G	G	G	A	A	A	G	C	G	A	C	G	G	T	G	A	G	A	T	C	T	A	T	C	C	C	T	T	A	A	G	C	G	G
Cluster13:1(1,0)	T	A	G	G	G	A	A	A	G	C	G	A	C	G	G	T	G	A	G	T	T	C	A	A	A	C	C	C	T	T	A	G	A	C	G	G
Cluster11:1(1,0)	T	R	R	G	G	A	A	A	G	C	G	W	Y	G	G	T	S	W	G	A	T	C	A	A	A	C	C	C	T	T	A	A	G	Y	G	G
Cluster26:1(1,0)	T	A	G	G	R	M	R	R	G	Y	G	W	Y	R	G	T	S	W	G	A	T	C	A	A	A	C	C	C	T	T	A	R	G	C	G	G
Cluster27:1(1,0)	Y	G	R	K	R	A	A	G	G	T	G	T	T	A	A	G	S	W	G	A	T	C	A	A	A	T	T	M	Y	C	M	G	G	Y	A	A

TS-201

PMR6 Solyc02g080910 SL2.50ch02 45010413 45015714

54.394.569; C>T; intron variant

Deleterious SNP

Haplotypes	0: 54392871	1: 54392911	2: 54392921	3: 54392978	4: 54393584	5: 54393855	6: 54393996	7: 54394484	8: 54394524	9: 54394569	10: 54394589	11: 54394602	
Cluster7:1(1,0)	G	G	C	A	C	G	A	T	C	T	T	T	TS-122
Cluster0:175(175,0)	G	G	C	A	C	G	A	T	C	C	T	T	
Cluster2:1(1,0)	G	G	C	R	C	G	A	T	C	C	T	T	
Cluster3:1(1,0)	G	G	C	A	C	G	A	T	C	C	W	T	
Cluster4:1(1,0)	G	G	C	A	C	G	A	Y	C	C	T	T	
Cluster5:1(1,0)	G	R	C	A	C	G	A	T	C	C	T	T	
Cluster9:1(1,0)	G	G	C	A	M	G	A	T	C	C	T	T	
Cluster6:1(1,0)	G	A	C	A	C	G	A	T	Y	C	T	T	
Cluster8:1(1,0)	G	G	A	A	A	A	G	T	C	C	A	C	
Cluster1:2(2,0)	C	A	C	A	A	G	G	T	T	C	A	C	

**PMR6**	**Solyc02g093580**	**SL2.50ch02**	**54392803**	**54394803**

62.344.382; C>T; stop gained

Deleterious SNP

Haplotypes	0: 62343411	1: 62343600	2: 62343750	3: 62343754	4: 62343780	5: 62343860	6: 62344382	7: 62344409	8: 62344436	9: 62344553	10: 62344585	11: 62344612	12: 62344621	13: 62344758	14: 62344826	15: 62344895	16: 62345045	
Cluster3:1(1,0)	A	C	A	G	T	C	T	A	C	T	T	C	G	A	T	G	C	TS-7
Cluster7:1(1,0)	T	T	A	G	T	C	T	A	Y	T	T	T	G	A	C	G	T	
Cluster1:1(1,0)	T	T	A	G	T	G	C	G	T	T	T	T	G	C	Y	G	T	
Cluster5:1(1,0)	T	T	A	G	T	G	C	G	T	T	T	T	G	M	C	G	T	
Cluster0:176(176,0)	T	T	A	G	T	G	C	G	T	T	T	T	G	C	C	G	T	
Cluster4:1(1,0)	T	T	G	G	A	G	C	A	T	T	C	C	G	C	T	G	T	
Cluster8:1(1,0)	T	T	G	G	W	G	C	R	T	T	Y	Y	G	M	Y	G	T	
Cluster2:1(1,0)	T	T	G	A	A	G	C	A	T	A	C	C	G	A	T	G	T	
Cluster9:1(1,0)	T	T	R	R	W	G	C	G	T	A	C	Y	G	A	T	G	T	
Cluster6:1(1,0)	T	T	A	G	A	G	C	G	T	T	T	T	A	C	T	A	T	

**PMR6**	**Solyc03g111690**	**SL2.50ch03**	**62343287**	**62345438**

43.625.286; A>T; stop gained

Deleterious SNP

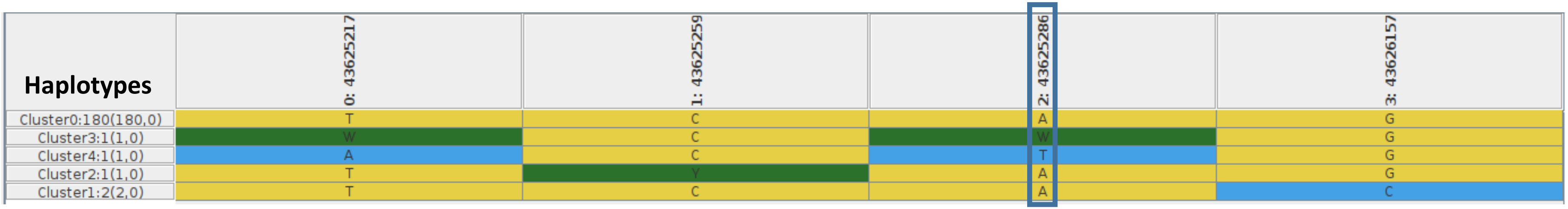

TS-68

**PMR6**	**Solyc06g071020**	**SL2.50ch06**	**43624823**	**43626842**
