# Supplementary material for: Genomic Analysis Highlights Putative Defective Susceptibility Genes in Tomato Germplasm
Source: Plants (Basel). 2023 Jun 12;12(12):2289. doi: 10.3390/plants12122289 (PMC10300993; doi:10.3390/plants12122289)
Supplement: Supplementary file 1 [file plants-12-02289-s001.zip › plants-2347108-Figure S2.pdf]

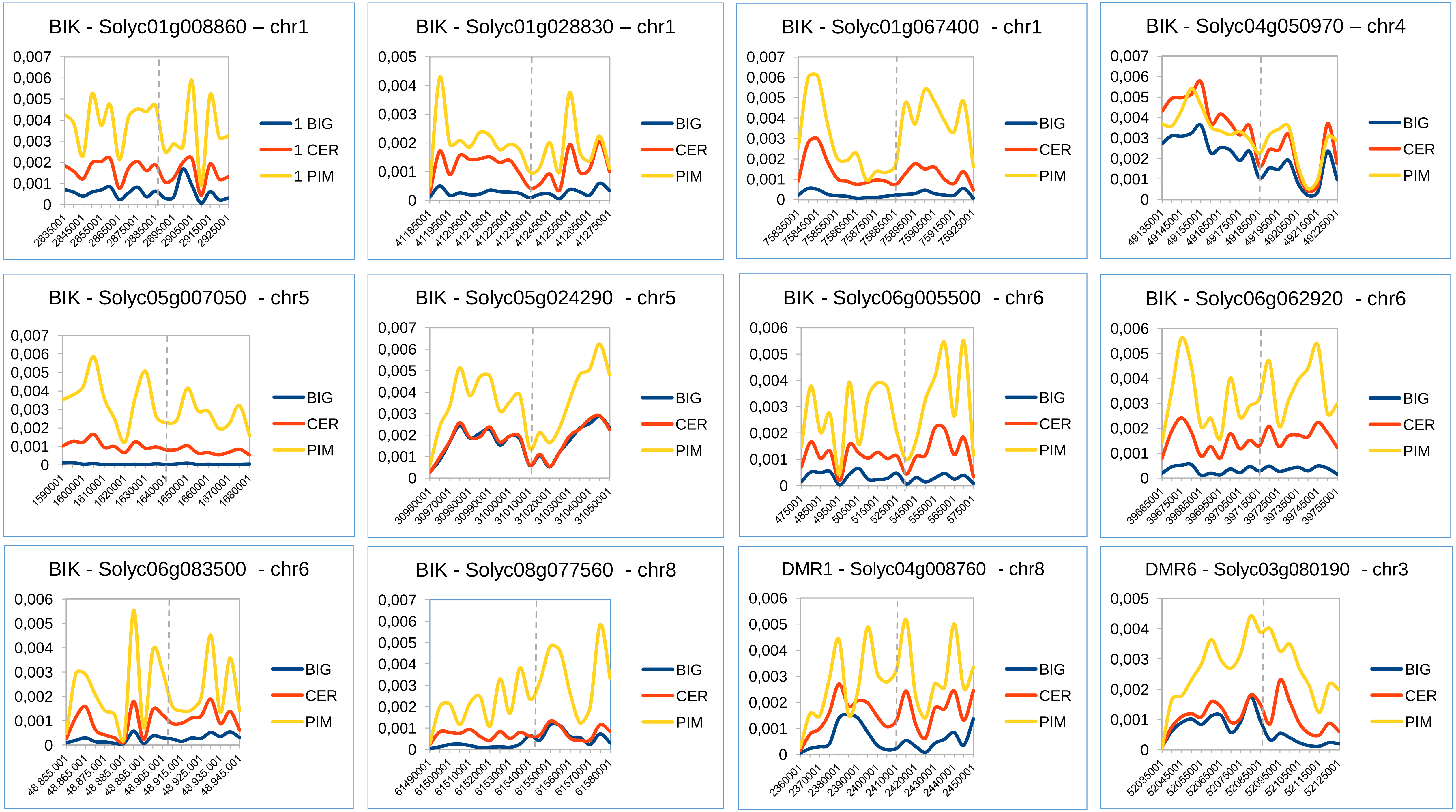
BIK - Solyc01g008860 – chr1
0,007 0,006 0,005 0,004 0,003 0,002 0,001 0
2835001 2845001 2855001 2865001 2875001 2885001 2895001 2905001 2915001 2925001
1 BIG
1 CER
1 PIM
BIK - Solyc01g028830 – chr1
0,005 0,004 0,003 0,002 0,001 0
41185001 41195001 41205001 41215001 41225001 41235001 41245001 41255001 41265001 41275001
BIG
CER
PIM
BIK - Solyc01g067400 - chr1
0,007 0,006 0,005 0,004 0,003 0,002 0,001 0
75835001 75845001 75855001 75865001 75875001 75885001 75895001 75905001 75915001 75925001
BIG
CER
PIM
BIK - Solyc04g050970 – chr4
0,007 0,006 0,005 0,004 0,003 0,002 0,001 0
49135001 49145001 49155001 49165001 49175001 49185001 49195001 49205001 49215001 49225001
BIG
CER
PIM
BIK - Solyc05g007050 - chr5
0,007 0,006 0,005 0,004 0,003 0,002 0,001 0
1590001 1600001 1610001 1620001 1630001 1640001 1650001 1660001 1670001 1680001
BIG
CER
PIM
BIK - Solyc05g024290 - chr5
0,007 0,006 0,005 0,004 0,003 0,002 0,001 0
30960001 30970001 30980001 30990001 31000001 31010001 31020001 31030001 31040001 31050001
BIG
CER
PIM
BIK - Solyc06g005500 - chr6
0,006 0,005 0,004 0,003 0,002 0,001 0
475001 485001 495001 505001 515001 525001 545001 555001 565001 575001
BIG
CER
PIM
BIK - Solyc06g062920 - chr6
0,006 0,005 0,004 0,003 0,002 0,001 0
39665001 39675001 39685001 39695001 39705001 39715001 39725001 39735001 39745001 39755001
BIG
CER
PIM
BIK - Solyc06g083500 - chr6
0,006 0,005 0,004 0,003 0,002 0,001 0
48.855.001 48.865.001 48.875.001 48.885.001 48.895.001 48.905.001 48.915.001 48.925.001 48.935.001 48.945.001
BIG
CER
PIM
BIK - Solyc08g077560 - chr8
0,007 0,006 0,005 0,004 0,003 0,002 0,001 0
61490001 61500001 61510001 61520001 61530001 61540001 61550001 61560001 61570001 61580001
BIG
CER
PIM
DMR1 - Solyc04g008760 - chr8
0,006 0,005 0,004 0,003 0,002 0,001 0
2360001 2370001 2380001 2390001 2400001 2410001 2420001 2430001 2440001 2450001
BIG
CER
PIM
DMR6 - Solyc03g080190 - chr3
0,005 0,004 0,003 0,002 0,001 0
52035001 52045001 52055001 52065001 52075001 52085001 52095001 52105001 52115001 52125001
BIG
CER
PIM

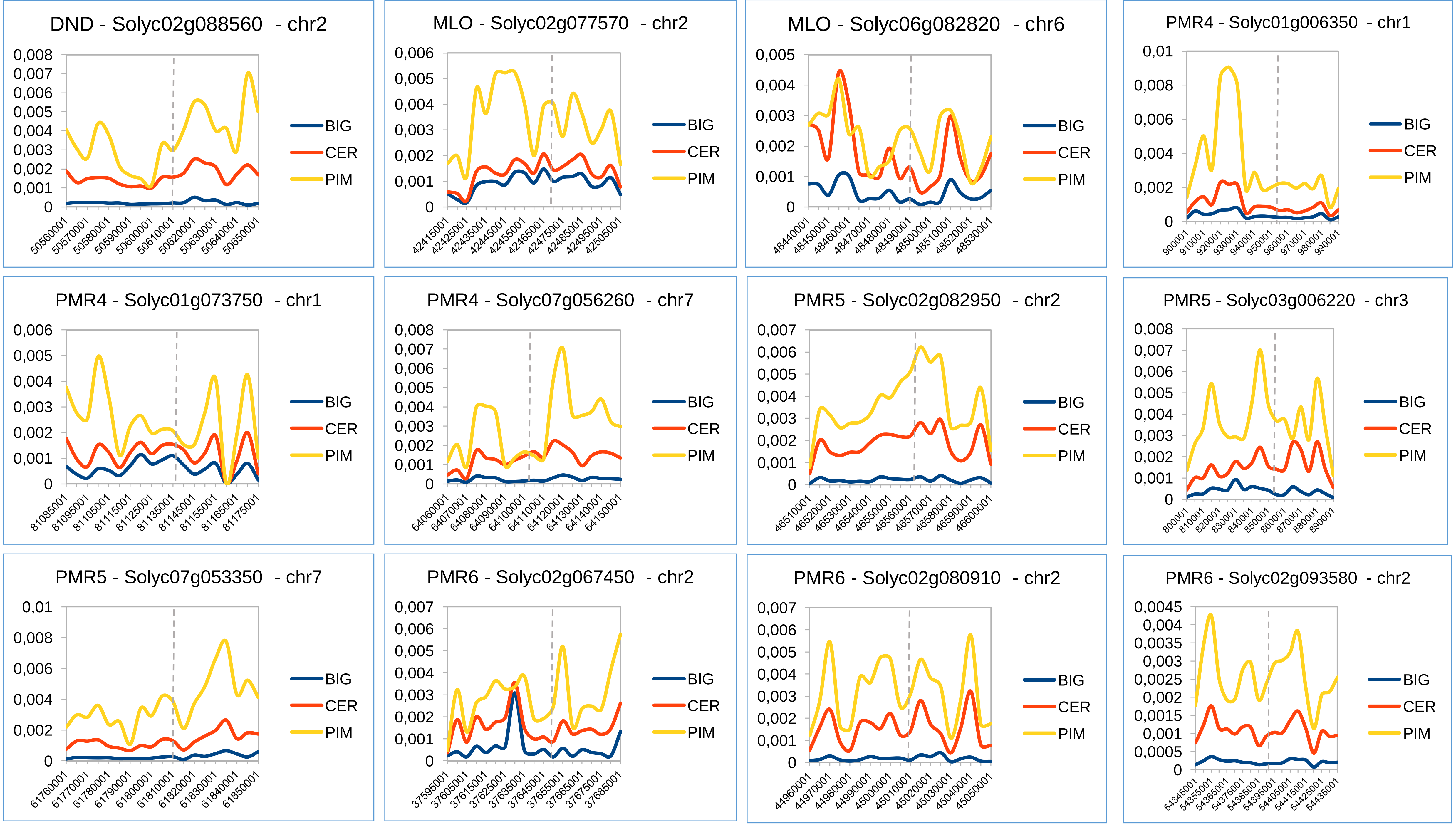
DND - Solyc02g088560 - chr2
0,008
0,007
0,006
0,005
0,004
0,003
0,002
0,001
0
50560001
50570001
50580001
50590001
50600001
50610001
50620001
50630001
50640001
50650001
BIG
CER
PIM
MLO - Solyc02g077570 - chr2
0,006
0,005
0,004
0,003
0,002
0,001
0
42415001
42425001
42435001
42445001
42455001
42465001
42475001
42485001
42495001
42505001
BIG
CER
PIM
MLO - Solyc06g082820 - chr6
0,005
0,004
0,003
0,002
0,001
0
48440001
48450001
48460001
48470001
48480001
48490001
48500001
48510001
48520001
48530001
BIG
CER
PIM
PMR4 - Solyc01g006350 - chr1
0,01
0,008
0,006
0,004
0,002
0
900001
910001
920001
930001
940001
950001
960001
970001
980001
990001
BIG
CER
PIM
PMR4 - Solyc01g073750 - chr1
0,006
0,005
0,004
0,003
0,002
0,001
0
81085001
81095001
81105001
81115001
81125001
81135001
81145001
81155001
81165001
81175001
BIG
CER
PIM
PMR4 - Solyc07g056260 - chr7
0,008
0,007
0,006
0,005
0,004
0,003
0,002
0,001
0
64060001
64070001
64080001
64090001
64100001
64110001
64120001
64130001
64140001
64150001
BIG
CER
PIM
PMR5 - Solyc02g082950 - chr2
0,007
0,006
0,005
0,004
0,003
0,002
0,001
0
46510001
46520001
46530001
46540001
46550001
46560001
46570001
46580001
46590001
46600001
BIG
CER
PIM
PMR5 - Solyc03g006220 - chr3
0,008
0,007
0,006
0,005
0,004
0,003
0,002
0,001
0
800001
810001
820001
830001
840001
850001
860001
870001
880001
890001
BIG
CER
PIM
PMR5 - Solyc07g053350 - chr7
0,01
0,008
0,006
0,004
0,002
0
61760001
61770001
61780001
61790001
61800001
61810001
61820001
61830001
61840001
61850001
BIG
CER
PIM
PMR6 - Solyc02g067450 - chr2
0,007
0,006
0,005
0,004
0,003
0,002
0,001
0
37595001
37605001
37615001
37625001
37635001
37645001
37655001
37665001
37675001
37685001
BIG
CER
PIM
PMR6 - Solyc02g080910 - chr2
0,007
0,006
0,005
0,004
0,003
0,002
0,001
0
44960001
44970001
44980001
44990001
45000001
45010001
45020001
45030001
45040001
45050001
BIG
CER
PIM
PMR6 - Solyc02g093580 - chr2
0,0045
0,004
0,0035
0,003
0,0025
0,002
0,0015
0,001
0,0005
0
54345001
54355001
54365001
54375001
54385001
54395001
54405001
54415001
54425001
54435001
BIG
CER
PIM

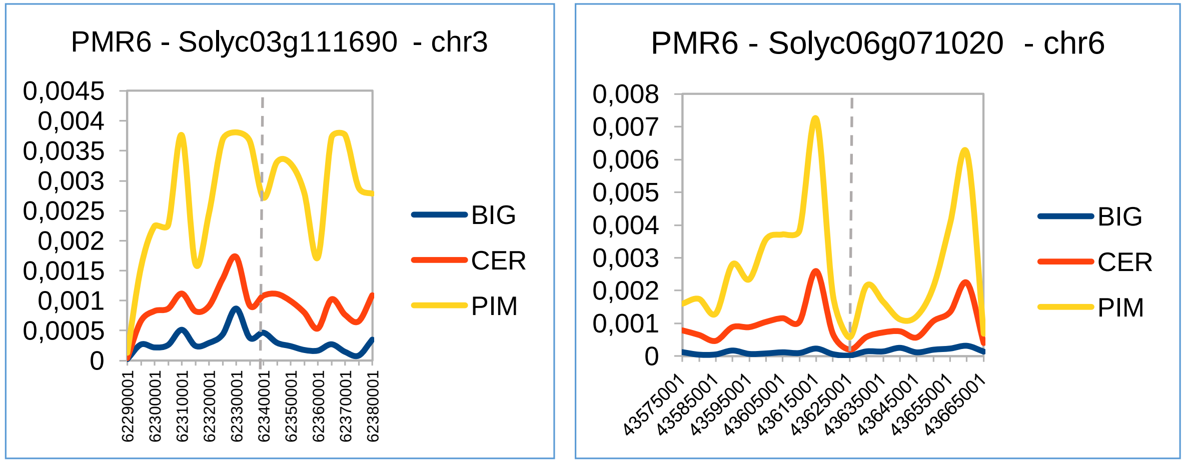

Figure S2. Nucleotide Diversity Analysis of S-Genes around Deleterious SNPs in PIM, CER, and BIG Groups. The genetic variation around of S alleles was analysed using the nucleotide diversity (Pi) index implemented in vcftools (https://vcftools.github.io), among different tomato groups (PIM, CER, and BIG), focusing on a 100kb region, centered around each deleterious SNP with a 5k window
