# Supplementary material for: Genomic Analysis Highlights Putative Defective Susceptibility Genes in Tomato Germplasm
Source: Plants (Basel). 2023 Jun 12;12(12):2289. doi: 10.3390/plants12122289 (PMC10300993; doi:10.3390/plants12122289)
Supplement: Supplementary file 1 [file plants-12-02289-s001.zip › plants-2347108-Figure S3.pdf]

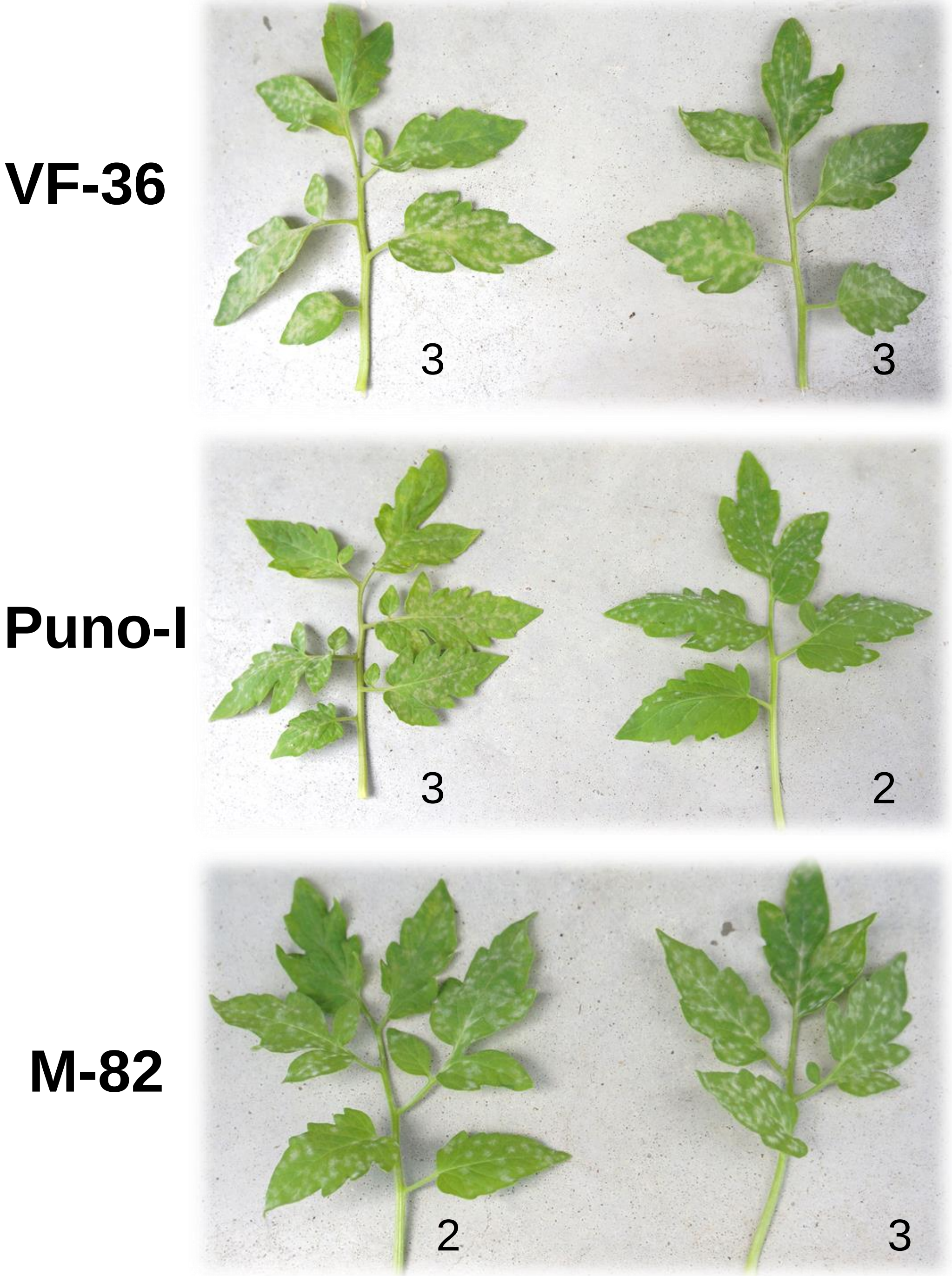

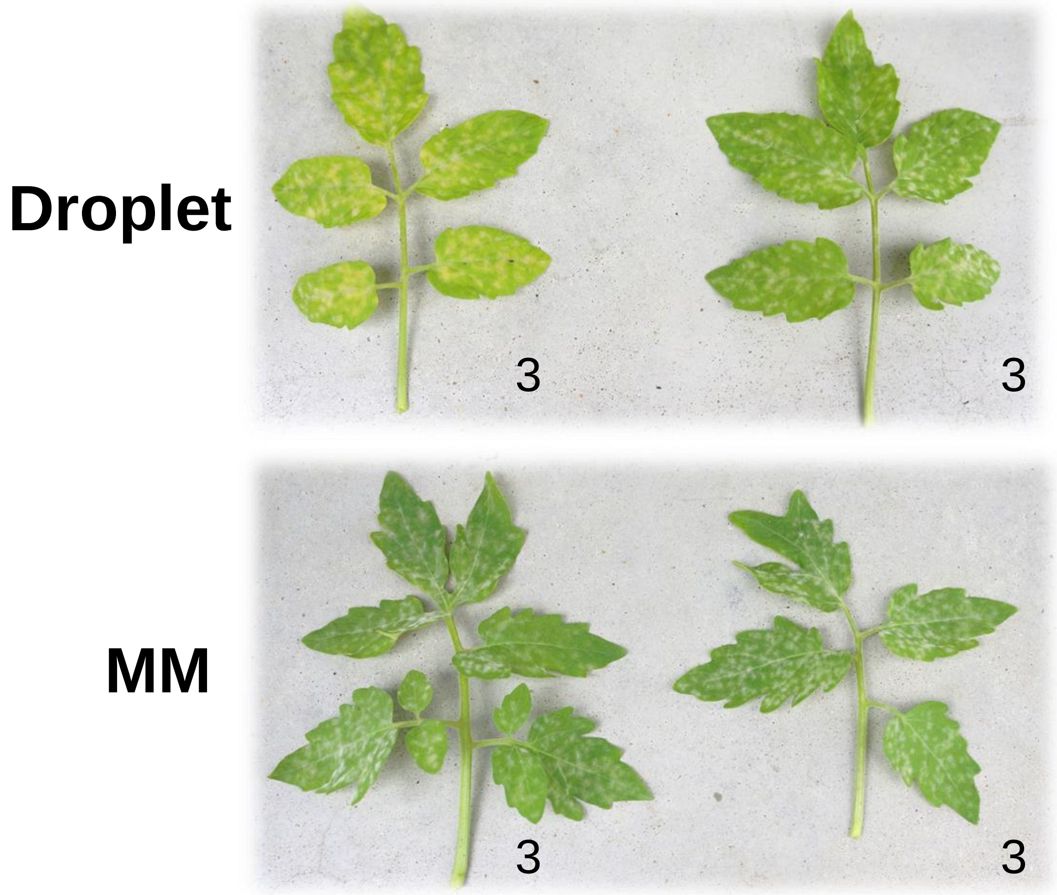

Figure S3. Example of results obtained with a pathogen assay performed with *Oidium neolycopersici* on four varieties (VF-36, M-82, Droplet, Puno-I) and a control variety (Moneymaker, MM) at 8 days post-inoculation (dpi). Disease scores were marked at the right corner of the leaves.
